# Reconstruction of Secondary Metabolic Pathway to Synthesize Novel Metabolite in *Saccharopolyspora erythraea*

**DOI:** 10.3389/fbioe.2021.628569

**Published:** 2021-07-02

**Authors:** Chong-Yang Ren, Yong Liu, Wen-Ping Wei, Junbiao Dai, Bang-Ce Ye

**Affiliations:** ^1^Institute of Engineering Biology and Health, Collaborative Innovation Center of Yangtze River Delta Region Green Pharmaceuticals, College of Pharmaceutical Sciences, Zhejiang University of Technology, Hangzhou, China; ^2^Laboratory of Biosystems and Microanalysis, State Key Laboratory of Bioreactor Engineering, East China University of Science and Technology, Shanghai, China; ^3^Guangdong Provincial Key Laboratory of Synthetic Genomics, Shenzhen Key Laboratory of Synthetic Genomics and Center for Synthetic Genomics, Shenzhen Institute of Synthetic Biology, Shenzhen Institutes of Advanced Technology, Chinese Academy of Sciences, Shenzhen, China

**Keywords:** CRISPR-Cas9, *Saccharopolyspora erythraea*, polyketide, Acyl-CoA, heterologous expression, metabolic pathway

## Abstract

Natural polyketides play important roles in clinical treatment, agriculture, and animal husbandry. Compared to natural hosts, heterologous chassis (especially Actinomycetes) have many advantages in production of polyketide compounds. As a widely studied model Actinomycete, *Saccharopolyspora erythraea* is an excellent host to produce valuable heterologous polyketide compounds. However, many host factors affect the expression efficiency of heterologous genes, and it is necessary to modify the host to adapt heterologous production. In this study, the CRISPR-Cas9 system was used to knock out the erythromycin biosynthesis gene cluster of Ab (erythromycin high producing stain). A fragment of 49491 bp in genome (from SACE_0715 to SACE_0733) was deleted, generating the recombinant strain AbΔ*ery* in which erythromycin synthesis was blocked and synthetic substrates methylmalonyl-CoA and propionyl-CoA accumulated enormously. Based on AbΔ*ery* as heterologous host, three genes, AsCHS, RgTAL, and Sc4CL, driven by strong promoters Pj23119, PermE, and PkasO, respectively, were introduced to produce novel polyketide by L-tyrosine and methylmalonyl-CoA. The product (*E*)-4-hydroxy-6-(4-hydroxystyryl)-3,5-dimethyl-2H-pyrone was identified in fermentation by LC-MS. High performance liquid chromatography analysis showed that knocking out *ery* BGC resulted in an increase of methylmalonyl-CoA by 142% and propionyl-CoA by 57.9% in AbΔ*ery* compared to WT, and the yield of heterologous product in AbΔ*ery*:AsCHS-RgTAL-Sc4CL was higher than WT:AsCHS-RgTAL-Sc4CL. In summary, this study showed that AbΔ*ery* could potentially serve as a precious heterologous host to boost the synthesis of other valuable polyketone compounds using methylmalonyl-CoA and propionyl-CoA in the future.

## Introduction

Knockout and cloning of long fragment DNA, especially those containing large gene clusters, is particularly important for synthetic biology and chemical biology research (El-Sayed et al., 2017; Shao et al., 2018). Although homologous recombination has been applied to knock out single or multiple genes in the genome, it is difficult to remove long fragment DNA sequences in the genome, such as biosynthetic gene clusters, before the emergence of CRISPR-Cas9 technology (Komor et al., 2016; Park et al., 2016; Su et al., 2016). For example, a new yeast strain with a single chromosome was created by using CRISPR-Cas9 that achieved the deletion of long redundant repetitive sequences in chromosome and the accuracy of chromosome fusions (Shao et al., 2019). And then CRISPR-Cas9 was applied in *Streptomyces* as a more efficient tool for genome editing (Cobb et al., 2015; Tong et al., 2015). Researchers established a highly efficient CRISPR-Cas9 genome editing plasmid pKCcas9dO for the genetic manipulation of *Streptomyces*, with an editing efficiency of 60–100%. The system has been applied for single gene deletions such as actII-orf4 *redD*, *glnR*, and knocking out large gene clusters such as antibiotic biosynthetic gene clusters (ACT, 21.3 kb), red pigment synthesis gene clusters (31.6 kb), and Ca^2+^-dependent antibiotics (82.8 kb) (Huang et al., 2015). The advances of genetic engineering tools and strategies accelerated the programs that introduce designed metabolic pathways in the strains for industrial production. Novel and efficient DNA splicing methods including BioBrick assembly (Storch et al., 2015), Gibson assembly (Gibson et al., 2009; Arturo et al., 2013), TAR clone (Ross et al., 2014), etc., facilitate multi-fragment, large gene cluster assembly (Zhang W. et al., 2017) and the manipulation of genes involved in metabolic pathways (Guo et al., 2015).

*Saccharopolyspora erythraea* is a Gram-positive bacterium and a model representative of Actinobacteria. It is widely used in industry for large-scale production of Erythromycin A (ErA), and has great value of research (Moffitt, 1998; Kim and Cerniglia, 2005). At the end of the last century, scholars discovered the location of erythromycin biosynthetic gene clusters, from SACE_0713 to SACE_0734, with a total length of about 56 kb and containing 21 erythromycin synthesis-related genes (Thompson et al., 1982; Tomich, 1988; Reeves et al., 1999). After the whole genome sequencing of *S. erythraea* finished in 2007 (Oliynyk et al., 2007), its genetic modification has become more convenient. Traditional genetic modification relies on its own homologous recombination machinery, and the genes are knocked in or out through homology arms mediated double exchange (Ferain et al., 1996; Tsai et al., 2011). However, it is difficult and inefficient to edit the gene in *S. erythraea* which has high GC content. With the emergence and continuous optimization of the CRISPR-Cas9 system, we have better tools for gene editing. In previous work, we have successfully applied CRISPR-Cas9 in *S. erythraea* (Liu W. et al., 2018; Liu et al., 2019).

Heterologous production of natural products has attracted more attention in terms of microbial technology and the discovery of new active compounds (Cleto and Lu, 2017; Liu Y. et al., 2018; Huo et al., 2019). Not only can it produce more valuable compounds and higher yield in more suitable heterologous hosts (Horbal and Luzhetskyy, 2016; Tan et al., 2017), but it can also dig out new compounds through biosynthetic engineering and metabolic engineering (Zhang et al., 2016; Lopatniuk et al., 2017; Reynolds et al., 2017). Due to the differences of transcription and metabolic regulation, precursor supply, etc. between different hosts, the yield of heterologous expression is not sufficient (Zhang J. J. et al., 2017; Horbal et al., 2018). Therefore, it can be increased by various methods such as medium optimization, precursor feeding, adding strong promoter (Cortina et al., 2012), and deleting some known metabolic biosynthetic gene clusters (Mao et al., 2017). There are abundant propionyl-CoA (PP-CoA) or methylmalonyl-CoA (MM-CoA) in *S. erythraea* which can be used to synthesize valuable compounds (Li et al., 2013; Karničar et al., 2016; Cho et al., 2018). Polyketides are widely found in bacteria, fungi, and plants and have a variety of biological activities, such as antibiotics (Erythromycin), immunosuppressants (Rapamycin) (Calne et al., 1989), anti-tumor (Doxorubicin), and insecticidal Agent (Nanchangmycin) (Sun et al., 2003), etc. In recent years, studies on polyketone compounds increased (Yu et al., 2012; Lim et al., 2016; Parvez et al., 2018). Chalcone synthase (CHS) (EC2.3.1.74), a plant-derived type III polyketide synthase, can use malonyl-CoA or MM-CoA and 4-coumaryl-CoA, 4-Hydroxyphenylpropionyl-CoA, or benzoyl-CoA as substrates to produce phlorizin and chalcone (Morita et al., 2001). Because of its broad substrate specificity, it has been widely expressed in heterologous hosts such as *Escherichia coli* (Wu et al., 2013) and *Saccharomyces cerevisiae* (Lyu et al., 2017; Wang et al., 2019).

**FIGURE 1:**
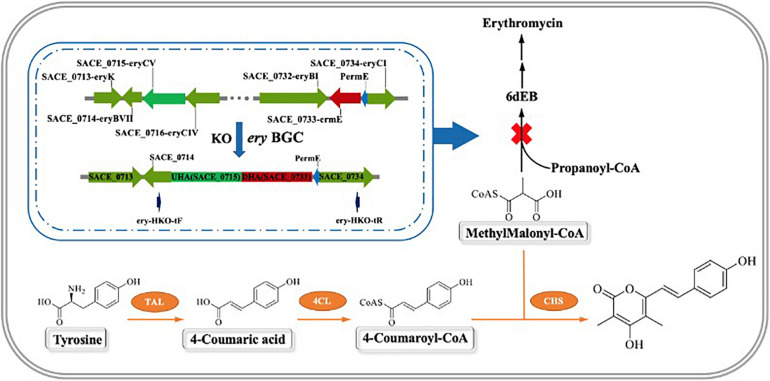
Schematic diagram of the reconstruction of secondary metabolic pathway and the synthesize route of novel metabolites BNY-type pyrone.

In this study, we constructed a temperature-sensitive plasmid pKECas9-erysgRNAII-HA, using two sgRNAs to specifically target SACE_0715 and SACE_0733 sites of the erythromycin biosynthetic gene cluster, and knocking out about 49.5 kb genomic sequence by providing a homologous repair template spanning the two targets, which blocked the synthesis of erythromycin. It was detected by HPLC that the erythromycin synthesis precursor MM-CoA was accumulated in a large amount compared with the WT. On this basis, a secondary metabolic pathway was constructed by introducing the heterologous genes CHS from *Aquilaria sinensis* (Gao et al., 2015), TAL from *Rhodotorula glutinis* (Wu et al., 2014), and 4CL from *Streptomyces coelicolor* (Hsiao and Kirby, 2008). These genes were constructed in integrative plasmid pSET152 by tandem, and driven by stronger promoters Pj23119, PermE, and PkasO to increase the heterologous expression. The route of research is shown in Figure 1, using L-tyrosine as the starting substrate and MM-CoA accumulated in the AbΔ*ery* to synthesize a new secondary metabolite (*E*)-4-hydroxy-6-(4-hydroxystyryl)-3,5-dimethyl-2H-pyrone (BNY-type pyrone), a kind of non-natural small molecule with novel structure catalyzed by plant polyketone polymerase. It can be used as a precursor to synthesize pyrone drugs and has a broad application prospect in the future (Abe et al., 2002; Gao et al., 2015).

## Materials and Methods

### Strains, Plasmids, and Growth Conditions

All recombinant strains and plasmids used in this study are listed in Supplementary Table 2. *E. coli* DH5α was used for construction of recombinant plasmids. *E. coli* were cultured at 30 or 37°C in Luria-Bertani broth (LB) medium (Tryptone 10 g/L, Yeast extract 5 g/L, NaCl 10 g/L), and apramycin (50 μg/mL) was added for plasmid cloning when required. In order to select the apramycin-resistant mutant strain of *S. erythraea* after transformation, 25 μg/mL or 50 μg/mL apramycin was used. The *S. erythraea* wild-type strain (NRRL23338), the erythromycin high-yield strain (Ab), and the AbΔ*ery* strain were grown on R2YE agar plates (Liao et al., 2015). For seed stock preparation, the strain was cultivated in a 250 mL flask containing 30 mL tryptic soy broth (TSB), shaken at 220 rpm for 48 h at 30°C. With the same culture conditions, 0.5 mL of the seed culture was inoculated into a 500 mL flask containing 100 mL of TSB medium with 0.5 g glycine, and strain samples were harvested for preparation of protoplast at the indicated time points (48 h). PEG-mediated transformations of protoplasts were performed as previously described (Liu Y. et al., 2018).

Recombinant plasmid construction was performed using a Hieff Clone Multi One Step Cloning Kit (Yeasen, Shanghai, China). Plasmid extraction was performed using an Endofree Mini Plasmid Kit II (Tiangen, Beijing, China). *S. erythraea* mutant was verified by colony polymerase chain reaction (PCR). Sequencing validation of all plasmid constructs (support information) was performed using Phanta Max SupperFidelity DNA polymerase (Vazyme, Nanjing China) and was confirmed by sequencing (Majorbio, Shanghai, China). Restriction enzymes, polymerases, and kits were used according to the supplier’s instructions (Takara, Japan).

### Construction of *ery* BGC Knock-Out and Heterologous Expression Plasmids

The analysis of sgRNA in the *ery* BGCs knock-out plasmid was referred to previous studies (Doench et al., 2014). Two sgRNAs were selected to target *ery* BGC and their transcription were driven by Pj23119 and PkasO promoters, respectively. The gRNA backbone was added to form sgRNAII, which was constructed into pUC57 vector for preparation.

The knock-out element was recombined and cloned into the *Xba*I + *Hin*dIII restriction site, and pKECas9 vector was used as a backbone (Liu Y. et al., 2018). The complete knock-out cassette consisting of *ery* BGC sgRNAII and the homology arms (KOery-UHA, KOery-DHA) flanking the target was obtained by overlapping PCR, and the cloning kit was used to construct the *ery* BGC knockout vector by the following steps: (1) The pUC57-erysgRNAII plasmid was used as a template to obtain the (*Xba*I) H-erysgRNAII-O fragment; the O-UHA-O and O-DHA-*Hin*dIII-H fragment were amplified from *S. erythraea* genomic DNA. (2) The (*Xba*I) H-erysgRNAII-O and O-UHA-O fragments were used to generated (*Xba*I) H-erysgRNAII-UHA-O by the first round of overlapping PCR. Then the (*Xba*I) H-erysgRNAII-UHA-O and O-DHA-*Hin*dIII-H were used to the second round of overlapping PCR, amplified to obtain the H-*Xba*I-erysgRNA-UHA-DHA-*Hin*dIII-H fragment. (3) The pKECas9 plasmid was digested with *Xba*I + *Hin*dIII to obtain the linear vector, homologous recombination with (*Xba*I) H-erysgRNAII-UHA-DHA-H (*Hin*dIII) fragment to construct the pKECas9-erysgRNAII-UHA-DHA plasmid (Supplementary Figure 3a). The positive clones were confirmed by PCR using the pKECas9-test-F, pKECas9-test-R primer pair.

The heterologous genes AsCHS, RgTAL, and Sc4CL were driven by the Pj23119 (Huang et al., 2015), PermE (Bibb et al., 1985), and PkasO (Wang et al., 2013; Phelan et al., 2017), respectively, and promoters were cloned into the *Xba*I + *Eco*RV site of the pSET152 vector. Firstly, three fragments including Pj23119-AsCHS commercially synthesized by Ruimian (Shanghai, China), PermE-RgTAL, and PkasO-Sc4CL maintained in our laboratory were amplified and their sequences were provided in Supporting Information. Next, Pj23119-AsCHS and PermE-RgTAL were fused by overlapping PCR to obtain Pj23119-AsCHS-PermE-RgTAL, then combining with PkasO-Sc4CL to generate (*Xba*I) H-Pj23119-CHS-PermE-TAL-PkasO-4CL-H (*Eco*RV). Finally, the (*Xba*I) H-Pj23119-AsCHS-PermE-RgTAL-PkasO-Sc4CL-H (*Eco*RV) linear fragment was cloned into pSET152 (*Xba*I, *Eco*RV) to complete construction, see schematic (Supplementary Figure 3b). Six positive clones were screened with M13 primer pairs and the No. 2 was selected for expansion culture to extract plasmid (Supplementary Figure 6a). Then corresponding primers were used to confirm that all three gene expression cassettes were present; the fragment Pj23119-AsCHS was 1235 bp, PermE-RgTAL was 2273 bp, and PkasO-Sc4CL was 1696 bp (Supplementary Figure 6b). The primers used to amplify DNA fragments are shown in Supplementary Table 3.

### AbΔ*ery* Erythromycin Bioactivity Analysis

Growth trends were analyzed by a microplate reader (BioTek Reader) (Liu et al., 2019). Cell density measurements at OD_600_ were acquired every 8 or 12 h and were analyzed using GraphPad Prism 7 software package (GraphPad Software). According to the previous method, the titer of erythromycin in AbΔ*ery* and control Ab fermentation were quickly analyzed by turbidimetry of antibiotic microorganisms. AbΔ*ery* and Ab were inoculated into 500 mL shake flasks containing 30 mL of ABPM8 industrial medium at 30°C, 220 rpm for 7 days. After fermentation was completed, broth was centrifuged at 12000 × *g*, 10 min, and the supernatant was extracted for subsequent analysis. *Bacillus subtilis* was inoculated in LB medium and cultured overnight at 37°C, 220 rpm, then transferred to new LB medium growth to OD_580_ was 0.4. Then, 200 μL above *B. subtilis* was added to a sterile 90 mm dish containing 20 mL of bioactivity assay medium (Supporting methods), then distributed to a 96-well cell culture plate by an 8-channel pipette, and each well was 135 μL. Adding 15 μl erythromycin standard and fermentation supernatant to the 96-well plate incubated at 37°C, 200 rpm for 2.5–3 h, set three replicates for each sample and measurement of OD_580_ was analyzed by microplate reader. Making sure the OD_580_ of blank control was not more than 0.5 and fitting the curve with OD_580_-logC to determine the linear range and standard curve.

The above fermentation was filtered through a 0.22 μm disposable organic phase filter, and the filtrate was accurately analyzed by HPLC to calculate the titer of erythromycin. The analysis conditions were following: solvent B phase (55% acetonitrile) and solvent A phase (1L Milli-Q H_2_O, 8.7 g K_2_HPO_4_, pH 8.2), the flow rate was 1.0 mL/min, and the column maintained at 40°C. UV spectra were acquired at 215 nm (Liu Y. et al., 2018). The peak area value and the standard concentration value were used to fit the standard curve, then the ErA titer of the sample was calculated.

### Determination of Three Intracellular CoAs Concentration

Intracellular PP-CoA and MM-CoA of AbΔ*ery*, Ab, and WT strains were extracted and assayed by HPLC. Strains were inoculated into 30 mL TSB medium at 30°C and cultured at 220 rpm for 48 h. The cells were washed twice with PBS and collected by centrifugation at 12000 × *g*, 10 min, then resuspended in 800 μL lysate (10% trichloroacetic acid and 90% 2 mM dithiothreitol). The mixture was freeze-thawed 2–3 times at –80°C and 4°C, centrifuged at 15,000 × *g* for 10 min, then the supernatant was transferred to an activated, equilibrated Sep-Pak column (1 ml, 50 mg tC18; Milford, MA, United States). After 3–5 min for adsorption, the column was washed with 1 ml ddH_2_O and eluted with 400 μL of 40% acetonitrile. SpeedVac (Thermo Fisher, Waltham, MA, United States) was used for lyophilization. It was dissolved by 100 μL of pure acetonitrile for HPLC analysis. The analytical conditions were mobile phase A (75 mM KH_2_PO_4_, pH 5.5) and mobile phase B (80% 75 mM aqueous KH_2_PO_4_, pH 5.0, mixed with 20% acetonitrile). Separation using a reversed-phase C18 column, flow rate 1 mL/min, column temperature 30°C, detector 254 nm, mobile phase distribution as follows: 4 min (when Buffer B reached 11% from 10%), 7 min (Buffer B reached 13% from 11%), 10 min (Buffer B reaches 15% from 13%), 15 min (Buffer B reaches 18% from 15%), 20 min (Buffer B reaches 23% from 18%), 23 min (Buffer B Results 28% from 23%), 28 min (Buffer B reaches 33% from 28%), 30 min (Buffer B reaches 39% from 33%), 50 min (Buffer B reaches 48% from 39%), 55 min (Buffer B reached 54% from 48%), and 65 min (Buffer B decreased from 54 to 10%), maintaining 10% phase B for 5 min, and stopping data collection at 70 min (Xu et al., 2017). Three independent experiments were performed to calculate the standard deviation.

### RNA Extraction and qRT-PCR Analysis

The strain was grown in 30 mL TSB medium to the late stage of the exponential phase, and collected by centrifugation at 12,000 × *g* for 10 min at 4°C. Total RNA was isolated and purified from the strain by RNA Prep Pure Cell/Bacteria Kit DP430 (TIANGEN) kit, and the RNA quality was assessed by 1% agarose gel electrophoresis and concentration was quantified by Synergy Mx multi-plate reader (BioTek, Winooski, VT, United States). Then, 1.0 μg total RNA with PrimeScript RT Reagent Kit and gDNA Eraser (Takara, Japan) kit were performed to synthesize cDNA which has removed genomic DNA. The resulting cDNA was diluted to final concentration of 50 ng/μL as the template, and real-time quantitative PCR analysis of mRNA levels was performed with 10 μL SYBR Premix Ex Taq GC (Takara) and the volume was 20 μL. All samples were prepared in triplicate to obtain CT values, and the relative gene expression levels were calculated using the comparative CT method (2^–ΔΔCt^) (Jinek et al., 2012; Liu et al., 2019). The qPCR assays were carried out by CFX96 Real-Time System (Bio-Rad) under conditions as following: 95°C for 5 min, then 40 cycles (95°C for 10 s, 60°C for 20 s, 72°C for 30 s), with a final extension cycle at 72°C for 10 min with *sigA* (SACE_1801) as the reference gene (Liao et al., 2015). The transcription levels of heterologous genes were determined by absolute quantification, and the copy number of constructed plasmid was diluted into gradient of 2 × 10^7^, 2 × 10^6^, 2 × 10^5^, 2 × 10^4^, 2 × 10^3^, 2 × 10^2^, 2 × 10^1^, 2 × 10^0^ as template for Q-PCR. Three replicates were set for each sample, and the copy number of heterologous genes were calculated by plotting the standard curve with the Ct value and the copy number logarithm as the horizontal and vertical coordinates. All primers in this work were described in Supplementary Table 3.

### HPLC and LC-MS Analysis of Fermentation

AbΔ*ery*:CT4, WT:CT4, AbΔ*ery*, and WT strains were transferred to a 250 mL flask containing 30 mL TSB medium and cultured at 37°C, 220 rpm for 7 days. The fermentations were centrifuged at 8000 × *g* to collect the supernatant, and concentrated. Then, 2 ml of the concentrate was filtered through a 0.22 μm disposable organic phase filter, and the filtrate was analyzed by HPLC. The analysis conditions were as follows: HPLC system (Waters 2695, water 2489 UV/visible light detector) equipped with a Thermo Hypersil BDS C18 column maintained at 35°C. The productions were analyzed at 277 nm, respectively, with mobile phase A (25 mM HCOONH_4_, pH 3.0) and mobile phase B (acetonitrile) at a flow rate 1.0 mL/min, mobile phase distribution as follows: 5 min (when Buffer B reached 10% from 2%), 20 min (Buffer B reached 40% from 10%), 25 min (Buffer B reached 2% from 10%). LC-ESIMS spectra were measured with a HPLC system (Agilent 1260) coupled to a 6530C Q-TOF LC-MS System (Agilent, Waldbronn, Germany). LC separation was carried out with a ZORBAX SB-C18 column (4.6 × 250 mm, 5 μm) at 30°C. For elution, H_2_O (solvent A) and acetonitrile (solvent B) were applied as the mobile phases at a flow rate of 1 mL/min. A gradient was used that the amount of solvent B as follows: 0–7 min (reached 60% from 5%), 9.5–12 min (reached 5% from 60%), 12–20 min (maintained 5%). The mass spectrometer was operated in the positive electrospray ionization (ESI) mode, the gas temperature and voltage were 300°C and 3.5 KV, nebulizer was 35 psig, collision energy 20 V.

## Results

### CRISPR-Cas9 Strategy for *ery* BGC Knock Out

**FIGURE 2:**
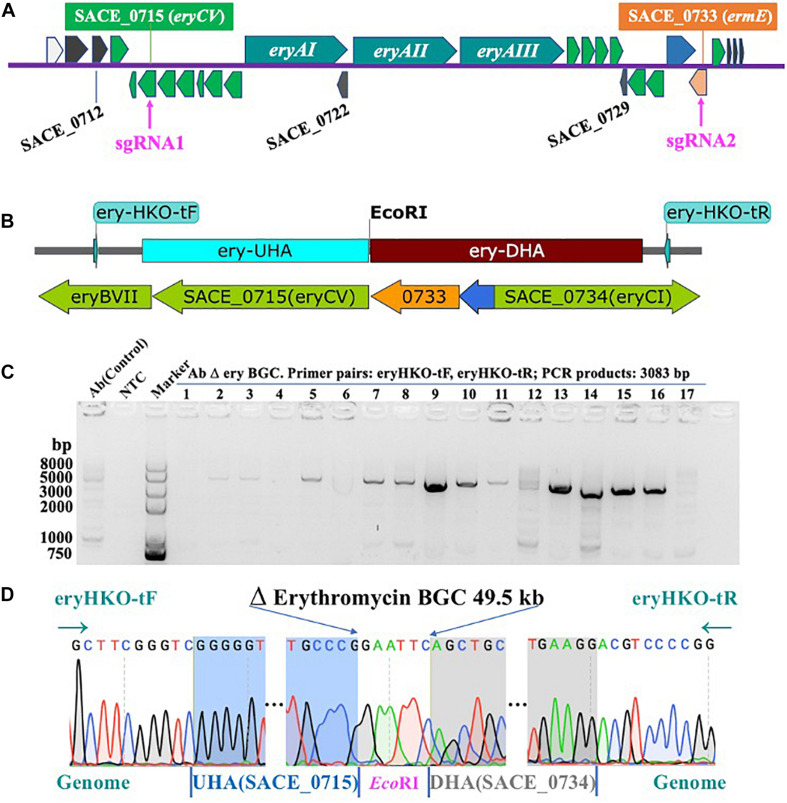
Knock-out of erythromycin biosynthetic gene cluster. **(A)** Erythromycin Biosynthetic Gene cluster (from SACE_0713 to SACE_0734) and the target of sgRNAs (SACE_0715 and SACE_0733). **(B)** Schematic of knock-out Erythromycin biosynthetic gene cluster. **(C)** Agarose gel electrophoresis of PCR products from AbΔ*ery* using test primer pairs (eryHKO-tF, eryHKO-tR). The size of PCR products is 3083 bp. **(D)** Sequencing of AbΔ*ery* BGC recombinant strain. 49491 bp sequence in *ery* BGC is knocked out.

Firstly, we used the Ape software to choose all sgRNAs that score more than 0.5 in sequence of *ery* BGC. In order to reduce the possibility of off-target as much as possible, two optimal sgRNAs were selected after using the BLAST function at the NCBI to confirm no sequence matches were found in the whole genome. The targets of the erythromycin biosynthesis gene cluster (*ery* cluster, SACE_0713-SACE_0734) were shown in Figure 2A, and provided details information of the entire *ery* BGC (Supplementary Figure 1 and Supplementary Table 1). The details of sgRNAs were shown in Table 1. Subsequently, we combined the two sgRNAs to sgRNAII under the control of Pj23119 and PkasO promoters, respectively, cloned into the PKEcas9 vector with the homology arms (KOery-UHA, KOery-DHA) on both sides of the target, and then the plasmid was transferred into *E. coli* DH5α. The primer pairs pKECas9-test-F, pKECas9-test-R were used to screen positive clones by colony PCR, and the results were shown in Supplementary Figure 2a. We expanded the positive clone and extracted plasmids for further verification using different primer pairs. The results of agarose gel electrophoresis indicated that the size of each band was correct (Supplementary Figure 2b) and the construction of plasmid pKECas9-erysgRNAII-HA was succeeded (Supplementary Figure 2c).

**TABLE 1:** Details of two sgRNAs for knock-out of *ery* BGC.

No.	*ery*-sgRNA (Seq.)	PAM	Loc	Dir	Score	5′G	3′GG	Matches	>15nt
1	GGCGAGGTCGGCGAGCCGGG	cGG	3513	>	0.692	0	1	1	1
2	GCACCGGCTTGAACAGCCGG	cGG	52743	>	0.856	1	1	1	1

*Seq, sgRNA sequence 5′ to 3′; Loc, location. Dir, direction, >indicates sgRNA on the sense strand, <indicates sgRNA on the antisense strand.*

After we transformed plasmid into Ab protoplasts, a single colony growing on the selective plates was randomly selected and subjected to PCR analysis to verify whether *ery* BGC has been knocked out. Successful deletion of the 49.5 kb DNA fragment will result in an amplification product at size 3083 bp; otherwise, no band should show up since it is too big to amplify. As shown in Figure 2C, among 17 clones, we found several PCR products at the right size. To further confirm, the PCR products were subjected to DNA sequencing. In Figure 2D, the sequencing results of No.9 showed that all core genes from SACE_0716 (*eryCIV*) to SACE_0732 (*eryBI*) in *ery* BGC were knocked out, and the 3′ end of the *eryCV* was 310 bp, and the 5′ end of *ermE* was 670 bp, which deleted 49491 bp sequence in *ery* BGC. In conclusion, using CRISPR-Cas9, we are able to successfully construct the *S. erythraea* strain in which the entire *ery* BGC has been removed (Figures 2B,D). This strain was designated AbΔ*ery* and cultured at 42°C several times, and lost the temperature-sensitive gene editing plasmid, which is convenient for subsequent research. Growth curves showed that knocked-out BGC reduced the metabolic pressure of AbΔ*ery* and made it grow better before 120 h, but biomass decreases slightly in later stages of culture (Supplementary Figure 4).

### Strain AbΔ*ery* Lost the Ability of Erythromycin Synthesis

**FIGURE 3:**
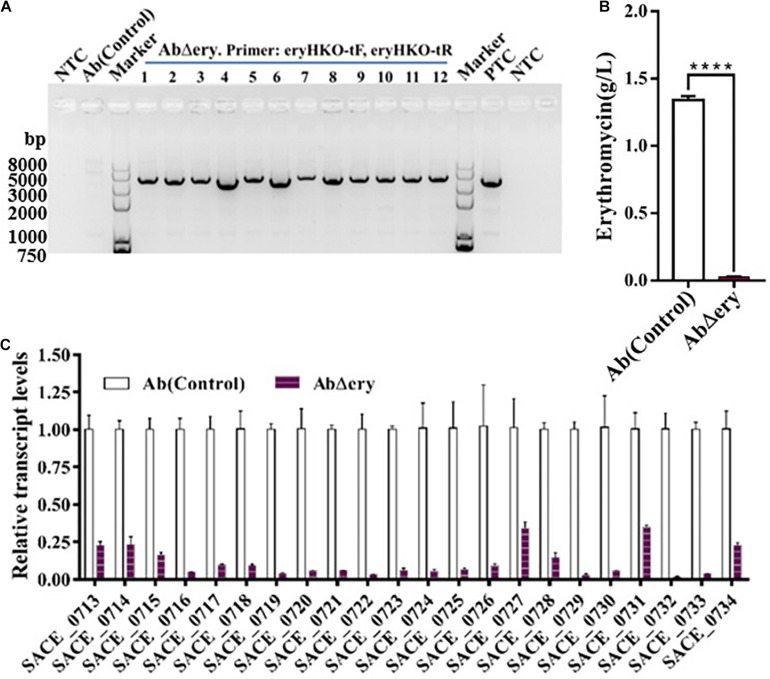
Strain AbΔ*ery* lost the ability of erythromycin synthesis. **(A)** Agarose gel electrophoresis of PCR products from subcultured AbΔ*ery* to screen single colony using test primer pairs (eryHKO-tF, eryHKO-tR). The PCR products is 3083 bp. **(B)** Production of ErA in Ab and AbΔ*ery* grown in fermentation medium. **(C)** Transcriptional analysis of erythromycin BGC expression of AbΔ*ery* and Ab at 48 h. Relative transcript levels were obtained individually after normalization to the *sigA* (SACE_1801) internal reference gene. Gene expression values observed in the control strain (Ab) were set as 1.0. Error bars indicate the standard deviations from three independent replicates.

In order to test whether deletion of *ery* BGC will lead to the loss of erythromycin synthesis, 12 colonies from the subcultured AbΔ*ery* strain were cultured (Figure 3A), and the biological activity method was used to determine the ability of erythromycin production with Ab as a control (Supplementary Table 8). The erythromycin synthesized by *S. erythraea* was not a single compound but included six isomers, ErA, ErB, ErC, ErD, ErE, and ErF, of which ErA was the highest antibacterial activity and the most widely used element. The biological activity method cannot determine the yield of ErA and the measurement results were not accurate which can only function as a reference, so the lowest yield AbΔ*ery* and Ab(control) were chosen and cultured in industrial fermentation medium for 7 days. Then the culture was sampled and centrifuged to collect the supernatant. The high-performance liquid chromatography (HPLC) was used to determine the yield of ErA (Figure 3B), which is nearly 1.5 g/L in Ab and barely detectable in AbΔ*ery*. Therefore, these data indicated that AbΔ*ery* has lost ability to synthesize ErA.

To further prove, we analyzed the transcription level of erythromycin biosynthetic gene cluster in AbΔ*ery*. The total RNA was extracted at 48 h and reverse-transcribed into cDNA as a template, after which the transcription level of *ery* BGC was determined by QPCR. The sigA (SACE_1801) was selected as reference gene and Ab was used as control. The primers designed from SACE_0713 to SACE_0734 in *ery* BGC were used to detect the transcription levels of each gene, information of primers in Supplementary Table 3. The results of the transcription level analysis were shown in Figure 3C and Supplementary Table 4, which indicate that the transcripts of genes for SACE_0713 to SACE_0734 were all downregulated. Furthermore, we found that a few gene transcriptions were still detected. The situation of contamination with wild type *S. erythraea* or cells that still contain *ery* BGC was ruled out by repeated experiments. We speculated that there were background signal errors or non-specific binding of primers and several genes such as SACE_0727 and SACE_0731 had multiple copies in genome of Ab compared to WT (Karničar et al., 2016), so there was still a small amount of transcription.

### Accumulation of MM-CoA and PP-CoA in AbΔ*ery* Strain

**FIGURE 4:**
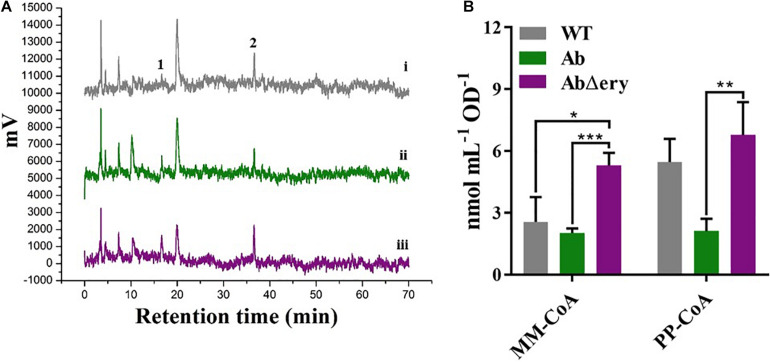
Determination of CoAs concentration by HPLC. **(A)** HPLC analysis of intracellular coenzyme A in WT (i), Ab (ii), and AbΔ*ery* (iii). The peak 1 is MM-CoA (methylmalonyl-CoA) which peak time in spectrum is 17.6 min, and 2 is PP-CoA (propionyl-CoA) which peak time is 35.9 min. **(B)** Comparison of peak area of MM-CoA and PP-CoA between AbΔ*ery* (purple), Ab (green), and WT (gray).

The biosynthesis of erythromycin is divided into two stages, the synthesis of 6-dEB (6-deoxyerythromycin-B) and post-synthesis modification. The 6-dEB was synthesized by a series of polyketide synthase using one unit of PP-CoA and six units of MM-CoA (Zhang J. J. et al., 2017). *S. erythraea* produced abundant MM-CoA and PP-CoA, which were used for the synthesis of erythromycin and maintaining own metabolism. When *ery* BGC was knocked out, synthesis of erythromycin will be blocked, and cells will accumulate a large amount of MM-CoA and PP-CoA. To verify that, we cultured AbΔ*ery*, Ab, and WT in TSB for 48 h before extracting intracellular CoAs and determining concentration by HPLC. We measured the contents of MM-CoA and PP-CoA in the recombinant strain. The HPLC results (Figure 4A and Supplementary Figure 5) showed that compared with WT and Ab, MM-CoA and PP-CoA were significantly accumulated in AbΔ*ery*. The concentration of MM-CoA in AbΔ*ery* was about 1.5 times of Ab (162%) and WT (142%), whereas the concentration of PP-CoA increased 220 and 57.9% than Ab and WT (Figure 4B).

### Heterologous Genes Were Introduced Into AbΔ*ery* to Reconstruct Secondary Metabolic Pathways

**FIGURE 5:**
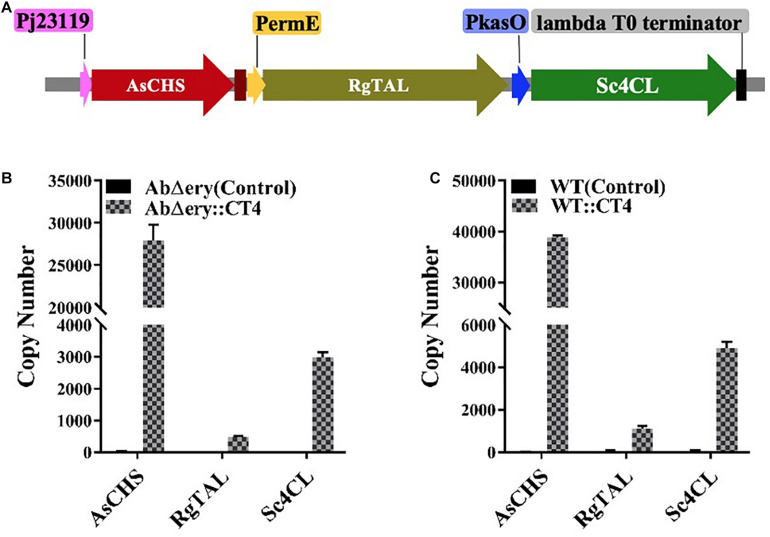
Confirm of pSET152-Pj23119-AsCHS-PermE-RgTAL-PkasO-Sc4CL plasmids. **(A)** Schematic diagram of heterologous genes expression cassette (Pj23119-AsCHS-PermE-RgTAL-PkasO-Sc4CL) in pSET152. **(B)** qRT-PCR analysis expression of AsCHS, RgTAL, and Sc4CL in AbΔ*ery*:CT4 with AbΔ*ery* as control. **(C)** qRT-PCR analysis expression of AsCHS, RgTAL, and Sc4CL in WT:CT4 with WT as control. Transcript levels were obtained individually after drawing a standard curve using Ct value and copy number logarithm as the horizontal and vertical coordinates with a serial copy number gradient plasmid as a control. Error bars indicate the standard deviations from three independent replicates.

The above experiments indicated that the mutant strain AbΔ*ery* was successfully constructed, in which the entire *ery* BGC has been deleted and the substrate CoAs were accumulated. Next, we would like to use AbΔ*ery* as a heterologous host to synthesize BNY-type pyrone using MM-CoA and L-tyrosine as substrates. RgTAL encodes tyrosine ammonia lyase which catalyzed L-tyrosine to 4-coumaric acid, Sc4CL encodes 4-coumarate-CoA ligase which synthesized to 4-coumaroyl-CoA, and AsCHS encoded chalcone synthase which catalyzed 1 unit of 4-coumaryl-CoA and 2 units of MM-CoA to BNY-type pyrone. In order to improve the efficiency of heterologous expression and ensure that AsCHS, RgTAL, and Sc4CL can be independently and completely expressed, they were driven by three strong promoters respectively, and constructed into the pSET152 vector (Figure 5A and Supplementary Figure 6d). According to the synthetic route, chalcone synthase catalyzing 4-coumaryl-CoA and MM-CoA to BNY-type pyrone was the rate-limiting step, therefore AsCHS was driven by the strongest promoter Pj23119, then Sc4CL was driven by PkasO and PermE for RgTAL. The sequencing results showed that the plasmid was successfully constructed (Supplementary Figure 6e).

The plasmid pSET152-Pj23119-AsCHS-PermE-RgTAL-PkasO-Sc4CL was transformed into AbΔ*ery* and WT protoplasts. Positive colonies AbΔ*ery*:AsCHS-RgTAL-Sc4CL (AbΔ*ery*:CT4), WT:AsCHS-RgTAL-Sc4CL(WT:CT4) were identified (Supplementary Figure 6c). The expression of heterologous genes was confirmed by reverse-transcription PCR using AbΔ*ery* and WT as control. The copy numbers of the three genes were calculated by absolute quantification (Figures 5B,C). This analysis showed that AbΔ*ery*:CT4 had the AsCHS copy number about 28000, the RgTAL copy number about 500, and the Sc4CL copy number about 3000, while WT:CT4 had the AsCHS copy number about 39000, the RgTAL copy number about 1100, and the Sc4CL copy number about 5000, the copy number of gene was consistent with the strength of its promoter. These data indicated that all genes were successfully transcribed.

### BNY-Type Pyrone Was Synthesized in Reconstructed Strain

**FIGURE 6:**
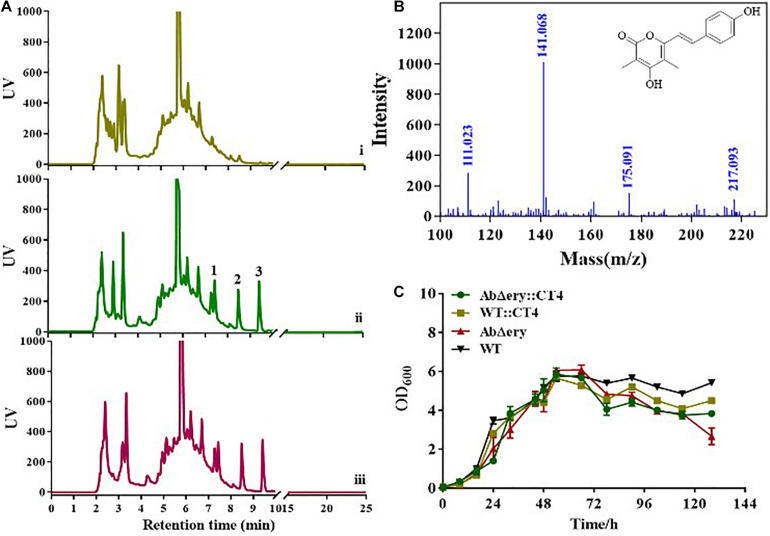
HPLC and LC-MS determination of heterologous gene expression products. **(A)** HPLC spectra of AbΔ*ery* **(i)**, WT:CT4 **(ii)**, and AbΔ*ery*:CT4 **(iii)**. Compared with control, three new peaks appeared after introduction of the heterologous genes, 1 is intermediate product 4-coumaric acid, 2 is the target product, and 3 is the by-product cinnamic acid (b) LC-ESIMS spectrum of 2 and the molecular ion peak [M + H]^+^ is m/z 259.0956. **(C)** Growth curve for the AbΔ*ery* (control), WT (control), AbΔ*ery*:CT4, and WT:CT4.

Agarose gel electrophoresis and QPCR analysis showed that three heterologous genes were successfully transferred into the AbΔ*ery* and had a high level of transcription. To further prove the role of heterologous genes, we analyzed the metabolites. AbΔ*ery*:CT4, WT:CT4, AbΔ*ery*, and WT were inoculated in TSB medium fermentation for 7 days, cultures were centrifuged to collect the supernatant, then it was concentrated by a freeze dryer and measured by HPLC (Figure 6A and Supplementary Figure 8a). The results showed that compared with the negative control, three new peaks were observed in the HPLC spectrum of AbΔ*ery*:CT4 and WT:CT4, and the peaks at 7.5 and 9.6 min were intermediate products 4-coumaric acid and cinnamic acid, 8.5 min was the target product, of which the yield in AbΔ*ery*:CT4 was much higher than WT:CT4. *In vitro* enzyme activity experiments showed that AsCHS can synthesize two products using MM-CoA and 4-coumaroyl-CoA, but it may be different *in vivo*. Therefore, we analyzed the structure of the target product by LC-MS, which the LC-ESIMS spectrum gave a molecular ion peak [M + H]^+^ at m/z 259 and was synthesized by two MM-CoA and 4-Coumaroyl-CoA (Figure 6B and Supplementary Figure 8c). Besides, their growth phenotypes were analyzed in TSB medium and growth curves showed that heterologous genes had no significant effect on their growth (Figure 6C). In summary, exogenous genes can play a role in AbΔ*ery*, and AbΔ*ery* can use the large amount of MM-CoA accumulated in the cell after knocking out *ery* BGC to produce more products, which has greater advantage than WT strains.

## Discussion

Heterologous expression of natural product biosynthetic pathways is widely used in synthetic biology field. It can not only dig hidden metabolites, identify the function of genome biosynthetic gene clusters (BGCs), but also enhance yield of valuable compounds in more suitable hosts (Huo et al., 2019). In addition to the level of expression of heterologous genes, the choice of host and its genetic background are critical for heterologous pathways to work (Zhang et al., 2010). By inhibiting competitive pathways, knocking out non-essential genes, or removing the toxicity of byproducts, the metabolic pathways of heterologous hosts can be altered, and precursor supply can be increased to achieve higher yields (Liu W. et al., 2018). The rapid development of molecular engineering technologies, including CRISPR-Cas9 and the advancement of synthetic biotechnology (Guo et al., 2015; Zhang W. et al., 2017), have effectively promoted the optimization of heterologous hosts.

Many secondary metabolites in actinomycetes belong to polyketones, which have a variety of biological activities. As a representative of engineering actinomycetes, *S. erythraea* has been widely studied, contains a large amount of PP-CoA and MM-CoA, and is suitable as a heterogeneous host for polyketides production. Therefore, we modified *S. erythraea* through chassis engineering, applied the CRISPR-Cas9 strategy to knock out large fragments of the genome to change its metabolic pathways. Two sgRNAs were designed to target SACE_0715 and SACE_0733 respectively. Q-PCR analysis of transcription level and HPLC analysis of fermentation showed that AbΔ*ery* lost the ability to synthesize erythromycin, and we found that the concentration of MM-CoA in AbΔ*ery* increased 220% and PP-CoA increased 162%. Therefore, AbΔ*ery* has advantages as a host to synthesize heterogenous polyketide.

For further verification, we introduced heterologous genes in AbΔ*ery* to reconstruct metabolic pathway. We selected type III polyketide synthase AsCHS, RgTAL, and Sc4CL, cloned into pSET152 plasmid, and used three constitutive strong promoters to enhance expression. New polyketide compounds were synthesized using MM-CoA and L-tyrosine as substrates in new metabolic pathway. The absolute quantification was used to analyze the transcription level of heterologous genes, the result showed all three genes were successfully expressed both in AbΔ*ery*:CT4 and WT:CT4. HPLC and LC-MS data showed that the yield of product in AbΔ*ery*:CT4 is higher than WT:CT4 (Figure 6A and Supplementary Figure 8b). Combined with the growth curve analysis, the biomass of AbΔ*ery* decreases faster than AbΔ*ery*:CT4 in later stages (Figure 6C). We speculate it may be due to blocking the *ery* BGC that initiates synthesis at the later stage, resulting in substrate accumulation. When the exogenous synthesis pathway is introduced, the substrate (PP-CoA and MM-CoA) accumulation pressure is reduced. Therefore, the growth of the reconstituted strain AbΔ*ery*:CT4 in the later stage returned to a state approximately to the original strain, and also produced more products. AbΔ*ery* have great advantages in production of polyketone compounds with MM-CoA as the substrate. Genome sequence analysis suggests that PCC pathway play a main role in providing MM-CoA and there are multiple loci such as SACE_0026-0028 and SACE_3398-3400 encode biotin-dependent carboxylases catalyzing carboxylation of PP-CoA to MM-CoA in *S. erythraea*. In future research, we want to enhance the metabolic pathway from PP-CoA to MM-CoA to improve the accumulation of MM-CoA. Besides, heterologous expression of other valuable polyketide such as spinosad which used both PP-CoA and MM-CoA as precursor in AbΔ*ery* is a feasible idea or activate silent gene clusters in its genome to discover new products.

In this study, we used the CRISPR-Cas9 system to achieve knock-out of long fragment gene clusters in *S. erythraea*, deleting the erythromycin biosynthesis gene clusters and accumulating precursor CoAs. Hence, *S. erythraea* became an excellent heterologous host that was beneficial for production of polyketides. We introduced heterologous genes type III polyketide synthase AsCHS, RgTAL and Sc4CL to reconstruct secondary metabolic pathway and synthesize new products which characterizes the ability of AbΔ*ery* as a heterologous host. Our results highlight the advantages of AbΔ*ery* as a heterologous host and the potential to produce more valuable exogenous polyketides.

## Data Availability Statement

The original contributions presented in the study are included in the article/Supplementary Material, further inquiries can be directed to the corresponding author/s.

## Author Contributions

C-YR and YL contributed equally, were responsible for experimental design, investigation, analysis, interpretation of data, and writing the original draft. JD and B-CY were responsible for the study’s conception and design, data analysis, and final approval of the manuscript. All the authors read and approved the final manuscript.

## Conflict of Interest

The authors declare that the research was conducted in the absence of any commercial or financial relationships that could be construed as a potential conflict of interest.
