# Supplementary material for: Reconstruction of Secondary Metabolic Pathway to Synthesize Novel Metabolite in Saccharopolyspora erythraea
Source: Front Bioeng Biotechnol. 2021 Jul 2;9:628569. doi: 10.3389/fbioe.2021.628569 (PMC8283810; doi:10.3389/fbioe.2021.628569)
Supplement: Supplementary file 1 [file Data_Sheet_1.docx]

Supplementary Information

**Reconstruction of Secondary Metabolic Pathway to Synthesize** **Novel Metabolites in *Saccharopolyspora erythraea***

Chong-Yang Ren ^1#^, Yong Liu ^2,3#^, Wen-Ping Wei ^2^, Junbiao Dai ^3^*, and Bang-Ce Ye ^1,2^*

[Author affiliations]

1Institute of Engineering Biology and Health, Collaborative Innovation Center of Yangtze River Delta Region Green Pharmaceuticals, College of Pharmaceutical Sciences, Zhejiang University of Technology, Hangzhou 310014, Zhejiang, China.

2Laboratory of Biosystems and Microanalysis, State Key Laboratory of Bioreactor Engineering, East China University of Science and Technology, Shanghai 200237, China.

3Guangdong Provincial Key Laboratory of Synthetic Genomics, Shenzhen Key Laboratory of Synthetic Genomics and Center for Synthetic Genomics, Shenzhen Institute of Synthetic Biology, Shenzhen Institutes of Advanced Technology, Chinese Academy of Sciences, Shenzhen 518055, China.

**List of Supplementary Figures and Tables**

Figure S1 Erythromycin Biosynthetic Gene Cluster (*ery* BGC)..............................................3

Figure S2 PCR Verification of DH5α/pKECas9-erysgRNAII-HA..........................................3
Figure S3 Construction of *ery* BGC knock-out and heterologous expression Vector.................4
Figure S4 Growth curve for the Ab (control) and Ab∆*ery*.....................................................4
Figure S5 HPLC analysis of MM-CoA and PP-CoA STD.....................................................5
Figure S6 Verification and transformation of heterologous expression Plasmid...........................6
Figure S7 Standard curve for absolute quantification of heterologous genes............................9
Figure S8 HPLC and LC-MS analysis of fermentation............................................................9

Table S1 Erythromycin Biosynthesis Gene Cluster.....................................................10

Table S2 Reagents, strains and plasmid used in this study..........................................11

Table S3 Primers used in this study.............................................................................12

Table S4 The data of *ery* BGC transcriptional analysis in Ab and Ab∆*ery*.................14

Table S5 Phanata Max Super-Fidelity PCR reaction system.......................................14

Table S6 Hieff One Step Cloning reaction system......................................................15

Table S7 Hieff PCR Master Mix reaction system.......................................................15

TABLE S8. Erythromycin Bioactivity Assay.............................................................15

**Materials and Methods
Plasmids, Strains and Cultivation Conditions**

Plasmids and Strains used in this study are listed in Table S1. *E. coli* DH5α was cultured in LB broth (10.0 g tryptone, 5.0 g yeast extract, 10.0 g NaCl, 1L H_2_O) at 37 °C, 220 rpm for plasmid construction. Solid media by adding 2.0 g/L agar to the media. Apramycin or kanamycin (50 µg/mL) were added to the media. *Saccharopolyspora erythraea* harboring plasmid were cultivated in TSB medium supplemented with 50 µg/mL apramycin or kanamycin.

**Erythromycin Bioactivity Assay**Make a preliminary judgment on the titer of the fermentation broth to guide the subsequent genetic engineering of the strain. Antibiotic microbiological tests include two methods, tube dish method and turbidity method, refer to the Chinese Pharmacopoeia 2015 edition 1201 chapter.

Media for Bioactivity assay

| **Components** | **Usage** |
| --- | --- |
| **LB Broth-250G** | 25.00g |
| **K_2_HPO_4_** | 3.68g |
| **KH_2_PO_4_** | 1.32g |
| **Glucose** | 1.00g |
| **Milli-Q H_2_O** | 1L |

The LB tube was inoculated with Bacillus subtilis 168, and cultured at 37 °C, 220 rpm shaker, until the OD_580_ was 0.4. Add 20 mL of bioactive assay medium to a sterilized 90mm culture dish, then add 200 μL of cultured *Bacillus subtilis* 168, mix and add to 96 well plate with 135 μL per well with an 8-channel pipette. Concentration gradient standards was added 15μL to per well (3 replicates). The fermentation broth sample was diluted, and the Ab high-yield industrial medium was fermented for 9 days and the sample was diluted (500, 1000, 2000 times, pre-experimentally determined dilution factor). The 96-well cell culture plate with the sample added was capped, cultured at 37 °C in an incubator at 200 rpm for 2.5-3 h, and the OD_580_ was measured with a microplate reader to control the blank control well OD_580_ to be no more than 0.5 (Liu et al., 2019).

**Supplementary Figures**


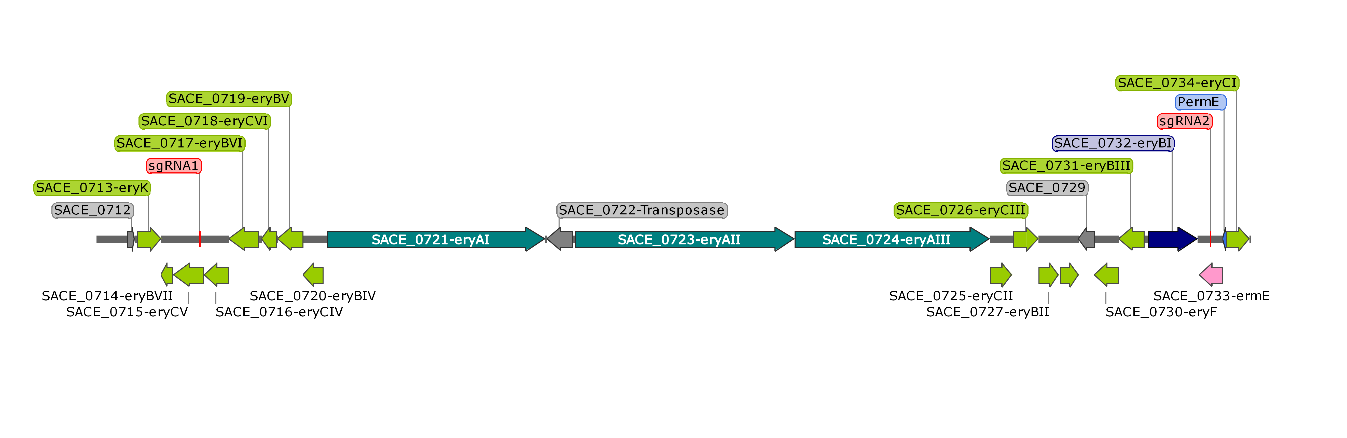
**FIGURE S1.** Erythromycin Biosynthetic Gene Cluster (*ery* BGC)


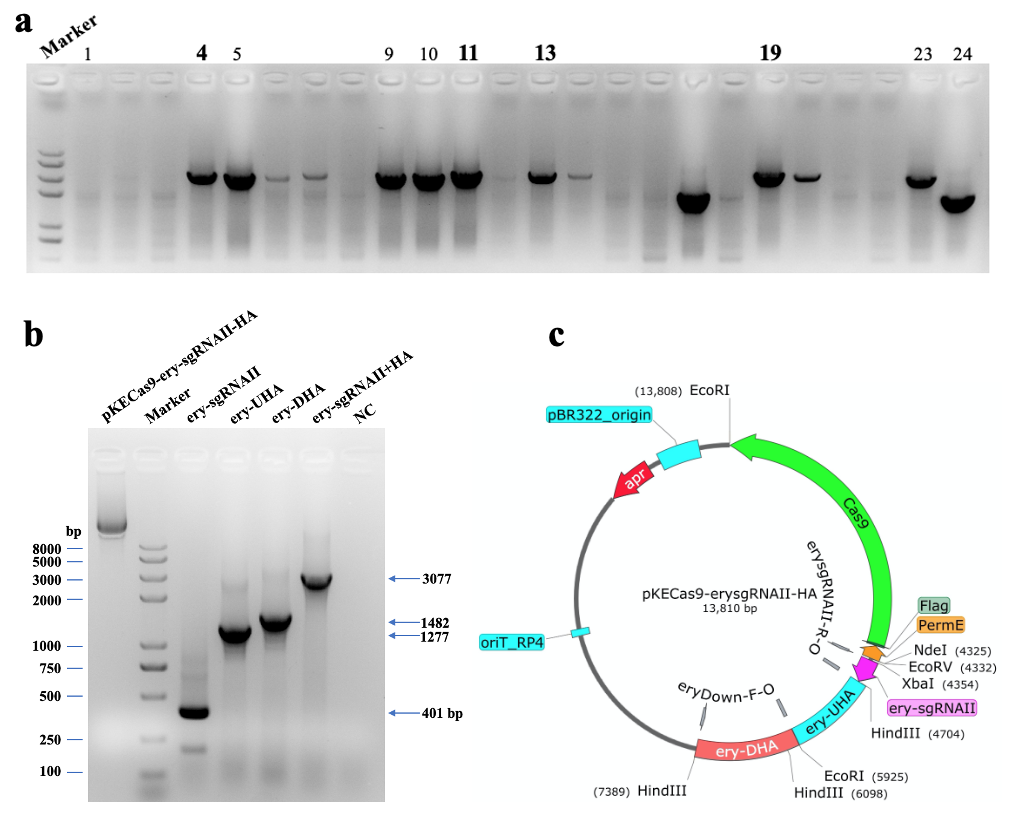


**FIGURE S2.** PCR verification of DH5α/pKECas9-erysgRNAII-HA.(a) Agarose gel electrophoresis of PCR products from DH5⍺/pKECas9-erysgRNAII-HA using test primer pairs. The PCR products of primer pair pKECas9-test-F, pKECas9-test-R is 3077 bp. (b) verification of pKECas9-erysgRNAII-HA plasmid. Lanes 1: pKECas9-erysgRNAII-HA plasmid; Lanes 2: Marker; Lanes 3: PCR products of erysgRNAII, size is 401 bp; Lanes 4: PCR products of ery-UHA, size is 1277 bp; Lanes 5: PCR products of ery-DHA, size is 1482 bp; Lanes 6: PCR products of erysgRNAII-UHA-DHA, size is 3077 bp; Lanes 7: negative control. (c) Map of pKECas9-erysgRNAII-HA. Overlapping PCR obtained sgRNAII sequence and ery-UHA, ery-DHA with homology arm, and then cloned into pKECas9(XbaI, HindIII) by one-step recombination.


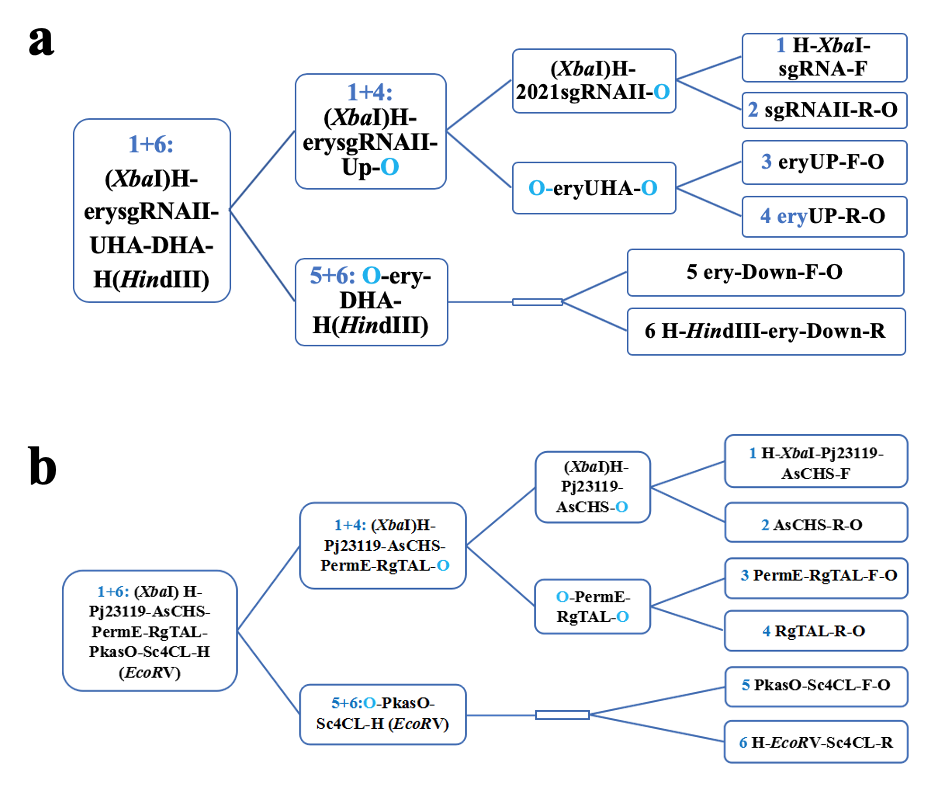


**FIGURE S3.** Construction of *ery* BGC knock-out and heterologous expression vector. (a) The step of construction (*XbaI*) H-*ery*sgRNAII-UHA-DHA-H (*HindIII*) fragment by Overlapping PCR. (b) The step of construction heterologous expression fragment by Overlapping PCR.


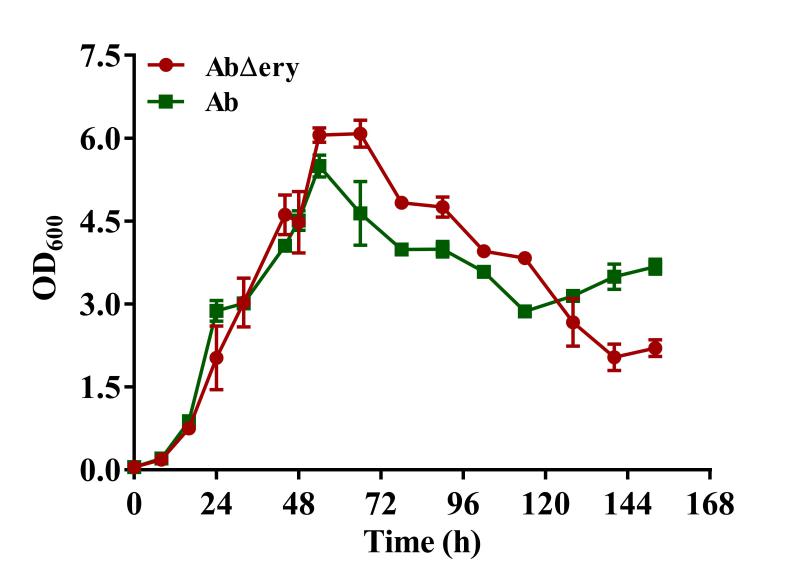


**FIGURE S4.** Growth curve for the Ab (control) and Ab∆*ery*.The cell optical density was measured by a microplate reader (BioTek Instruments, Winooski, VT, USA) in a 96-well microplate (Fluotrac 200; Greiner, Germany) with a wavelength of 600 nm.

**
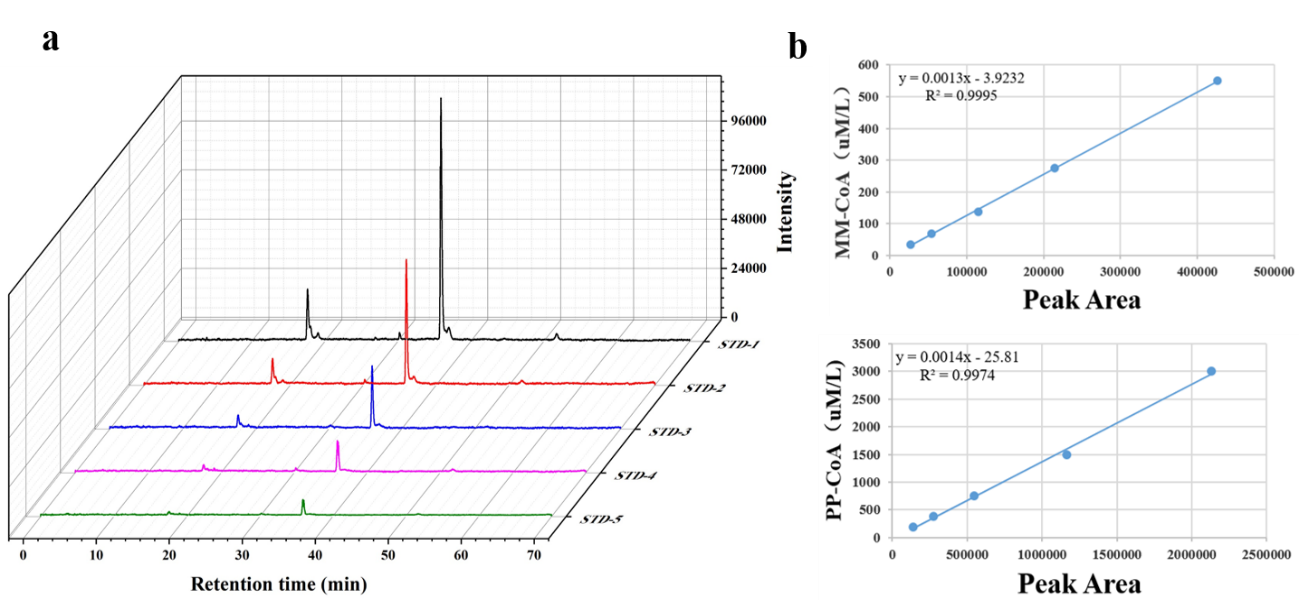
**

**FIGURE S5.** HPLC analysis of MM-CoA and PP-CoA STD. (a) HPLC analysis of MM-CoA (methylmalonyl-CoA) STD1-5 which concentration were 0.55, 0.275, 0.1375, 0.06875, 0.034375 mol/L and peak time was 17.6 min. The concentration of PP-CoA (propionyl-CoA) STD1-5 were 3.0, 1.5, 0.75, 0.375, 0.1875 mol/L and peak time was 35.9 min. (b) Standard curve for MM-CoA and PP-CoA.


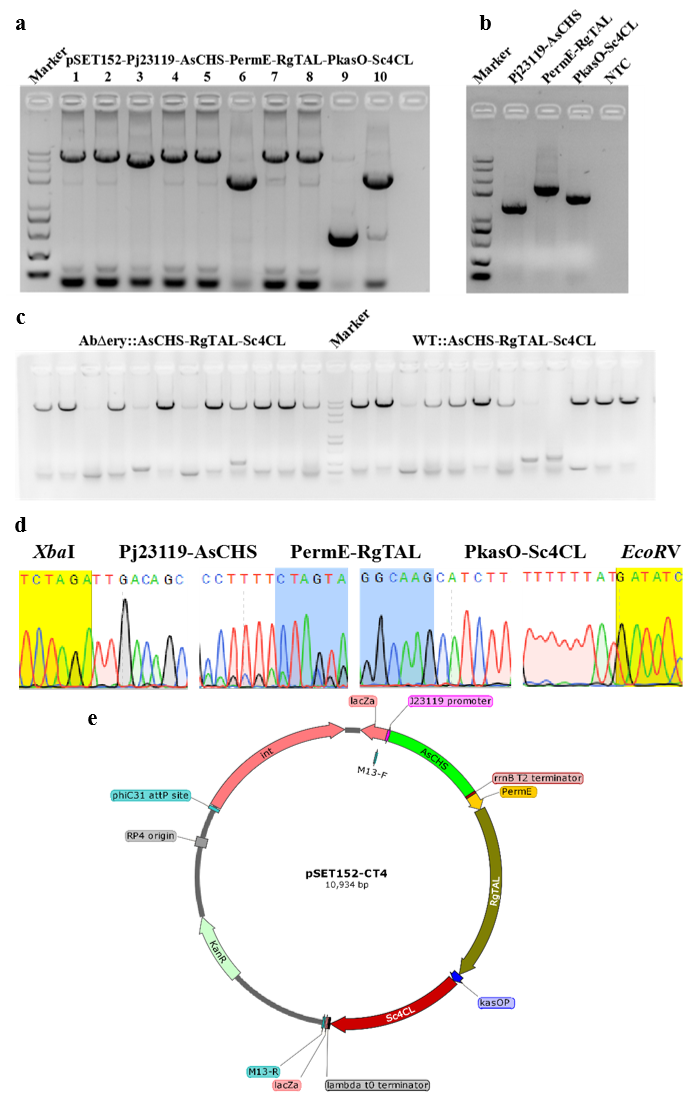


**FIGURE S6.** Verification and transformation of heterologous expression plasmid.(a) PCR-based identification of DH5⍺/pSET152-Pj23119-AsCHS-PermE-RgTAL-PkasO-Sc4CL using M13 primer pairs and the size of products is 5355 bp. (b) Verification of the fragments Pj23119-AsCHS, PermE-RgTAL and PkasO-Sc4CL by PCR in No.2 positive clone. (c) Transform plasmid into Ab∆*ery* and WT, positive colonies Ab∆*ery*::AsCHS-RgTAL-Sc4CL and WT::AsCHS-RgTAL-Sc4CL was confirmed by using M13 primer pairs. (d) The sequencing results of pSET152-CT4 (Pj23119-AsCHS-PermE-RgTAL-PkasO-Sc4CL) plasmid. (e) Map of pSET152-Pj23119-AsCHS-PermE-RgTAL-PkasO-Sc4CL plasmid, the size is 10934 bp.

**> Pj23119-AsCHS** (Gao et al., 2015; He et al., 2015)

ttgacagctagctcagtcctaggtataatgctagcATGGCCGCCAAAGTAGAAGAAATTCGCAAAGCCCGCCGCGCCGAAGGCCCAGCCACCGTACTAGCCATTGGCACCGCCGTACCAAACAACTGCCTACTACAAGCCGACTACCCAGACTACTACTTTCGCGTAACCAACAGCGAACACATGACCGAACTAAAAGAAAAATTTAAACGCATGTGCGACAAAAGCATGATTAAAAAACGCTACATGCACGTAACCGAAGAAGTACTAAAAGAAAACCCAAGCATGGCCGACTACTGGAGCCCAAGCCTAGACGCCCGCCAAGACATTGTAGTAGTAGAAATTCCAAAACTAGGCAAAGAAGCCGCCCAAAAAGCCATTAAAGAATGGGGCCAACCAAAAAGCAAAATTACCCACGTAGTATTTTGCACCACCAGCGGCGTAGACATGCCAGGCGCCGACTACCAACTAACCAAACTACTAGGCCTACGCCCAAGCGTAAAACGCCTAATGATGTACCAACAAGGCTGCTTTGCCGGCGGCACCGTACTACGCCTAGCCAAAGACCTAGCCGAAAACAACAAAGGCGCCCGCGTACTAGTAGTATGCAGCGAAATTACCGCCGTAACCTTTCGCGGCCCAAGCGAAACCCACCTAGACAGCATGGTAGGGCAGGCCCTATTCGGCGACGGAGCCGCCGCCATTATTGTAGGCAGCGACCCAGACACCCGCATTGAACGCCCACTATTTGAACTAATTAGCGCCGCCCAAACCATTCTACCAGACAGCGACGGCGCCATTGACGGCCACCTACGCGAAGTAGGCCTAACCTTTCACCTACTAAAAGACGTACCAGGCCTAATTAGCAAAAACATTGAAAAAAGCCTAGTAGAAGCCTTTACCCCAATTGGCATTAGCGACTGGAACAGCATTTACTGGATTGCCCACCCAGGCGGCCCAGCCATTCTCGACCAAGTAGAACAAAAACTAGGCCTAAAACAAGAAAAACTACGCGCCACCCGCCACATTCTAAGCGAATACGGCAACATGAGCAGCGCCTGCGTACTATTTATTCTCGACGAAATGCGCAAAAAAAGCCTAGAAGAAGGCAAAGCCACCACCGGCGAAGGCCTAGAATGGGGCGTACTATTTGGCTTTGGCCCAGGCCTAACCGTAGAAACCGTAGTACTACACAGCGTAGCCACCGAAAGCGCCCACTAAagaaggccatcctgacggatggcctttt

**> PermE-RgTAL** (Bibb et al., 1985; Wu et al., 2014)

ctagtatgcatgcgagtgtccgttcgagtggcggcttgcgcccgatgctagtcgcggttgatcggcgatcgcaggtgcacgcggtcgatcttgacggctggcgagaggtgcggggaggatctgaccgacgcggtccacacgtggcaccgcgatgctgttgtgggcacaatcgtgccggttggtaggatccaATGGCTCCTCGACCTACTTCGCAGTCGCAGGCTCGAACTTGTCCTACTACCCAGGTTACTCAGGTGGACATCGTGGAGAAGATGCTCGCCGCCCCTACTGACTCTACCCTTGAGCTGGACGGATACTCTCTCAACCTTGGTGACGTTGTTAGTGCGGCTCGAAAGGGTAGACCCGTGCGAGTGAAGGACTCGGACGAGATCCGATCTAAGATTGACAAGTCCGTTGAGTTCCTGCGATCGCAGCTGTCTATGTCGGTTTACGGCGTTACTACCGGATTCGGCGGTAGTGCTGACACTCGAACTGAGGACGCCATTTCGCTTCAGAAGGCTCTCCTGGAGCACCAGCTTTGCGGCGTCCTGCCTTCCTCGTTCGACTCGTTCCGACTGGGTCGAGGACTGGAGAACTCGCTCCCCCTTGAGGTTGTGCGAGGCGCCATGACCATCCGAGTGAACTCCCTTACACGGGGACACTCGGCTGTGCGACTGGTTGTGCTTGAGGCTCTCACTAACTTCCTTAACCACGGTATCACCCCTATTGTGCCTCTGCGAGGCACTATCTCCGCCTCGGGTGACCTGTCGCCTCTTTCCTACATTGCCGCTGCCATTTCGGGTCACCCCGACTCCAAGGTGCACGTTGTGCACGAGGGCAAGGAGAAGATTCTGTACGCCCGAGAGGCTATGGCCCTTTTCAACCTGGAGCCCGTGGTTCTGGGTCCTAAGGAGGGTCTGGGCCTTGTGAACGGAACCGCCGTGTCAGCTAGTATGGCTACTCTGGCTCTGCACGACGCCCACATGCTTTCGCTCCTGTCGCAGTCTCTTACCGCTATGACCGTTGAGGCGATGGTGGGACACGCCGGATCGTTCCACCCTTTCCTGCACGACGTCACCCGACCTCACCCTACTCAGATTGAGGTTGCCGGAAACATCCGAAAACTCCTTGAGGGATCGCGATTCGCCGTGCACCACGAGGAGGAGGTTAAGGTTAAGGACGACGAGGGTATACTGCGACAGGACCGATACCCCCTGCGAACTTCTCCCCAGTGGCTTGGTCCTCTGGTGTCTGACCTTATTCACGCCCACGCTGTTCTTACTATCGAGGCCGGTCAGTCCACCACTGACAACCCTCTTATTGACGTGGAGAACAAGACTTCGCACCACGGCGGAAACTTCCAGGCCGCTGCCGTGGCCAACACTATGGAGAAAACCCGACTGGGACTTGCCCAGATTGGTAAGCTCAACTTCACCCAGCTCACTGAGATGCTTAACGCCGGTATGAACCGAGGTCTTCCCTCGTGTCTTGCTGCTGAGGACCCCTCCCTGTCGTACCACTGTAAGGGTCTGGACATTGCCGCTGCTGCCTACACTTCTGAGCTGGGACACCTGGCTAACCCCGTCACTACTCACGTTCAGCCCGCTGAGATGGCTAACCAGGCCGTCAACTCCCTGGCTCTTATCTCCGCCCGACGAACCACTGAGTCCAACGACGTCCTGTCTCTTCTCCTTGCTACCCACCTTTACTGTGTTCTTCAGGCTATTGACCTGCGAGCCATTGAGTTCGAGTTCAAGAAGCAGTTCGGTCCCGCTATTGTTTCCCTCATTGACCAGCACTTCGGATCGGCCATGACCGGCTCGAACCTCCGAGACGAGCTTGTGGAGAAGGTGAACAAGACTCTTGCCAAGCGACTGGAGCAGACTAACTCGTACGACCTTGTTCCCCGATGGCACGACGCTTTCTCGTTCGCTGCCGGAACCGTTGTGGAGGTGCTTTCGTCTACTTCCCTGTCCCTTGCCGCTGTTAACGCTTGGAAGGTTGCTGCCGCTGAGTCGGCTATTTCTCTTACTCGACAGGTGCGAGAGACTTTCTGGTCGGCAGCCTCTACCTCGTCGCCTGCTCTGTCGTACCTTTCTCCCCGAACCCAGATTCTGTACGCTTTCGTGCGAGAGGAGCTTGGCGTCAAGGCCCGACGAGGTGACGTTTTCCTGGGCAAGCAGGAGGTTACCATCGGATCTAACGTGTCTAAGATTTACGAGGCTATCAAGTCCGGTCGAATTAACAACGTGCTTCTTAAGATGCTTGCCTAG

**> PkasO-Sc4CL** (Hsiao and Kirby, 2008; Wang et al., 2013)

tgttcacattcgaacggtctctgctttgacaacatgctgtgcggtgttgtaaagtcgtggccaggagaatacgacagcgtgcaggactgggggagttATGTTTCGTAGCGAATATGCAGATGTTCCGCCTGTTGATCTGCCGATTCATGATGCAGTTCTGGGTGGTGCAGCAGCATTTGGTAGCACACCGGCACTGATTGATGGCACCGATGGTACAACCCTGACCTATGAACAGGTTGATCGTTTTCATCGTCGTGTTGCAGCAGCCCTGGCAGAAACCGGTGTTCGTAAAGGTGATGTTCTGGCACTGCATAGCCCGAATACCGTTGCATTTCCGCTGGCATTTTATGCAGCAACCCGTGCCGGTGCAAGCGTTACCACCGTTCATCCGCTGGCCACCGCAGAAGAATTTGCAAAACAGCTGAAAGATAGCGCAGCACGTTGGATTGTTACCGTTAGTCCGCTGCTGAGCACCGCACGTCGTGCAGCAGAACTGGCAGGCGGTGTTCAAGAAATTCTGGTTTGTGATAGCGCACCGGGTCATCGTAGCCTGGTTGATATGCTGGCAAGTACCGCACCGGAACCGAGCGTTGCAATTGATCCGGCAGAAGATGTTGCAGCCCTGCCGTATAGCAGCGGCACCACCGGCACCCCGAAAGGTGTTATGCTGACCCATCGTCAGATTGCAACCAATCTGGCACAGCTGGAACCGAGTATGCCGAGTGCTCCGGGTGATCGTGTTCTGGCCGTTCTGCCGTTTTTTCATATTTATGGTCTGACCGCACTGATGAATGCACCGCTGCGTCTGGGTGCAACCGTTGTTGTTCTGCCTCGTTTTGATCTGGAACAGTTTCTGGCAGCAATTCAGAATCATCGTATTACCAGCCTGTATGTTGCACCGCCTATTGTTCTGGCGCTGGCAAAACATCCTCTGGTTGCAGATTATGATCTGAGCAGCCTGCGTTATATTGTTAGCGGTGCAGCTCCGCTGGATGCACGTCTGGCAGCCGCATGTAGCCAGCGCCTGGGTCTGCCTCCTGTTGGTCAGGCCTATGGCATGACCGAACTGAGTCCGGGTACACATGTTGTTCCTCTGGATGCAATGGCAGATGCACCGCCAGGCACCGTTGGTCGTCTGATTGCAGGTACAGAAATGCGTATTGTGAGCCTGACCGATCCGGGTACGGATCTGCCTGCGGGTGAAAGCGGTGAAATTCTGATTCGTGGTCCGCAGATCATGAAAGGTTATCTGGGTCGTCCGGATGCAACCGCAGCAATGATTGATGAAGAAGGTTGGCTGCATACCGGTGATGTTGGTCATGTTGATGCCGATGGTTGGCTGTTTGTTGTTGATCGTGTGAAAGAACTGATCAAATACAAAGGTTTTCAGGTGGCACCAGCCGAACTGGAAGCACATCTGCTGACACATCCGGGTGTTGCAGATGCAGCAGTTGTTGGTGCCTATGATGATGATGGTAATGAAGTTCCGCATGCCTTTGTTGTTCGTCAGCCAGCAGCTCCGGGTCTGGCCGAAAGCGAAATTATGATGTATGTGGCAGAACGTGTGGCACCGTATAAACGTGTTCGTCGTGTGACCTTTGTTGATGCGGTTCCGCGTGCAGCAAGTGGTAAAATTCTGCGTCGTCAGCTGCGTGAACCGCGTTAATAAttcagaacgctcggttgccgccgggcgttttttat


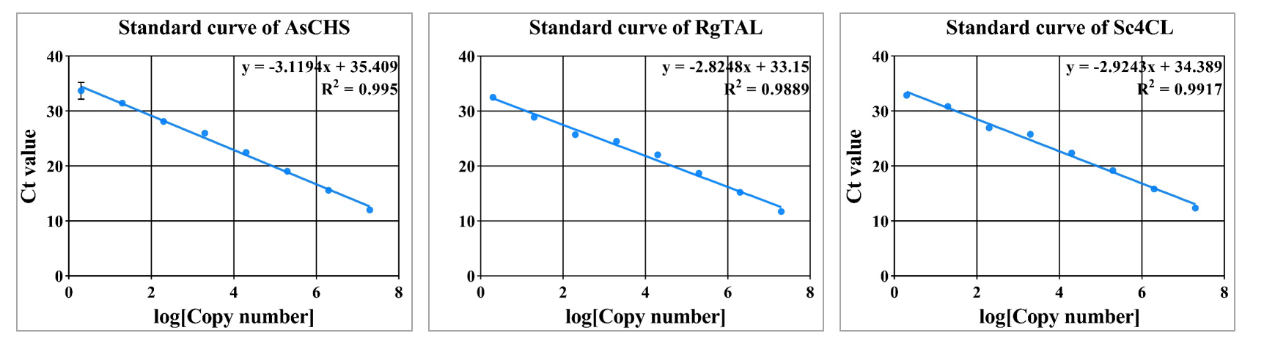
**FIGURE S7.** Standard curve for absolute quantification of heterologous genes.

**
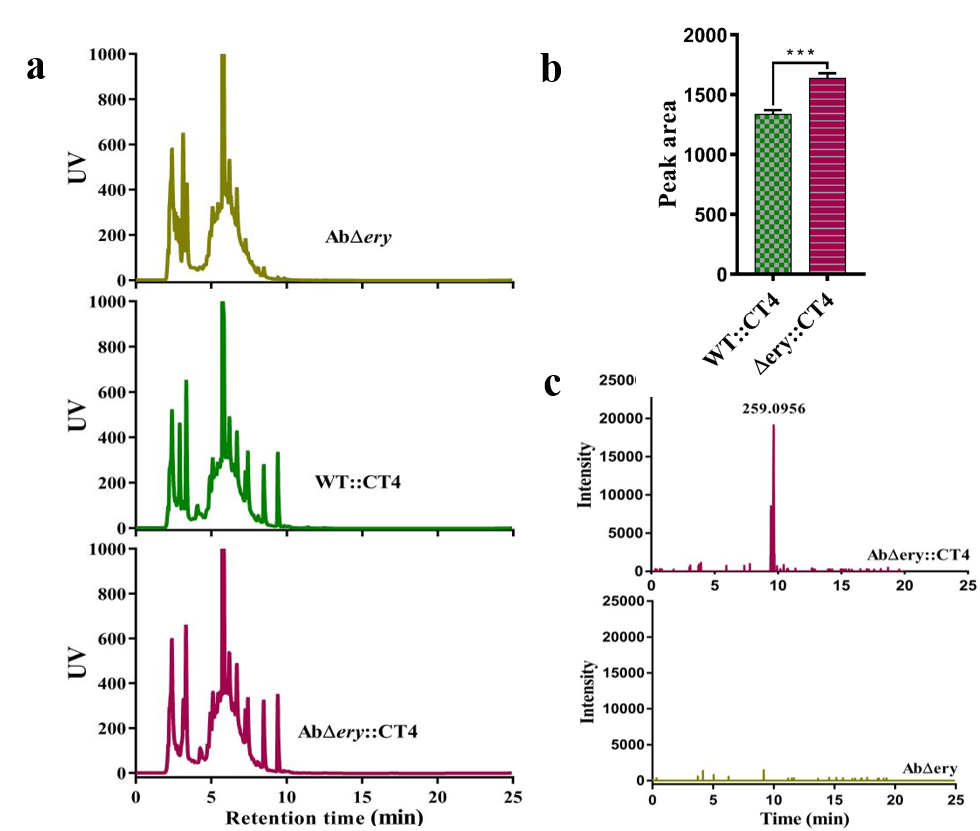
**

**FIGURE S8.** HPLC and LC-MS analysis of fermentation.(a) HPLC spectra of AbΔ*ery*, WT::CT4 and AbΔ*ery*::CT4. Compared with control, three new peaks appeared after introduction of the heterologous genes, which the peak time of intermediate product 4-coumaric acid is 7.5 min, the peak time of target product is 8.5 min and the peak time of by-product cinnamic acid is 9.5 min. (b) Peak area of target product in WT::CT4 and AbΔ*ery*::CT4 .(c) LC-ESI spectrum of target product and the molecular ion peak [M+H]^+^ is m/z 259.0956.

**Supplementary Tables**

**TABLE S1.** Erythromycin Biosynthesis Gene Cluster.

| **SACE** | **Gene** | **Dir** | **Enzyme Name** |
| --- | --- | --- | --- |
| 0712 | / | → | putative erythromycin esterase |
| 0713 | *eryK* | → | erythromycin 12-hydroxylase |
| 0714 | *eryBVII* | ← | dTDP-4-deoxyglucose 3,5-epimerase |
| 0715 | *eryCV* | ← | dTDP-4-amino-4,6-dideoxy-D-glucose ammonia-lyase |
| 0716 | *eryCIV* | ← | *eryCIV* NDP-6-deoxyhexose 3,4-dehydratase |
| 0717 | *eryBVI* | ← | TDP-4-keto-6-deoxy-D-glucose 2,3-dehydratase |
| 0718 | *eryCVI* | ← | TDP-desosamine-N-dimethyltransferase |
| 0719 | *eryBV* | ← | 6-DEB TDP-mycarosyl glycosyltransferase |
| 0720 | *eryBIV* | ← | dTDP-4-keto-6-deoxy-L-hexose 4-reductase |
| 0721 | *eryAI* | → | 6-deoxyerythronolide-B synthase, modules 1 and 2 |
| 0722 | / | ← | transposase |
| 0723 | *eryAII* | → | 6-deoxyerythronolide-B synthase, modules 3 and 4 |
| 0724 | *eryAIII* | → | 6-deoxyerythronolide-B synthase, modules 5 and 6 |
| 0725 | *eryCII* | → | TDP-4-keto-6-deoxy-glucose 3,4-isomerase |
| 0726 | *eryCIII* | → | 3-alpha-mycarosylerythronolide B desosaminyl transferase |
| 0727 | *eryBII* | → | TDP-4-keto-6-deoxyhexose 2,3-reductase |
| 0728 | *eryG* | → | erythromycin C methlytransferase |
| 0729 | / | ← | thioesterase |
| 0730 | *eryF* | ← | 6-deoxyerythronolide B hydroxylase |
| 0731 | *eryBIII* | ← | NDP-4-keto-2,6-dideoxyhexose 3-C-methyltransferase |
| 0732 | *eryBI* | → | beta-D-glucosidase |
| 0733 | *ermE* | ← | *ermC* 23S rRNA methyltransferase |
| 0734 | *eryCI* | → | dTDP-3-amino-3,4,6-trideoxy-alpha-D-glucose transaminase |

**Note**: Dir, Direction, → indicates gene on the sense strand, ← gene on the antisense strand.

**TABLE S2.** Reagents, strains and plasmid used in this study.

| **Reagent or resouce** | **Source** | **Identifier** |
| --- | --- | --- |
| **Chemicals and kits** |  |  |
| MiniBEST Agarose Gel Extraction Kit Ver.4.0 | Takara (Beijing) | 9762 |
| MiniBEST DNA Fragment Purification Kit Ver.4.0 | Takara (Beijing) | 9761 |
| DNA Ligation Kit Ver.2.1 | Takara (Beijing) | 6022 |
| PrimeScript RT ragent Kit with gDNA Eraser | Takara (Beijing) | RR047A |
| SYBR Premix Ex Taq GC | Takara (Beijing) | RR071A |
| Phanta Max Supper-Fidelity DNA Polymerase | Vazyme (Nanjing) | P505 |
| Hieff Clone^TM^ Multi One Step Cloning Kit | Yeasen | 10906ES10 |
| RNAprep pure Cell/Bacteria Kit | TIANGEN | #DP430 |
| Endofree Mini Plasmid Kit II | TIANGEN | #DP118-02 |
| *EcoR*I | Takara | 1040S |
| *EcoR*V | Takara | 1042S |
| *Hind*III | Takara | 1060S |
| *Xba*I | Takara | 1093S |
| *Nde*I | Takara | 1161A |
| **Experimental models: organisms/strains** |  |  |
| *Escherichia coli* DH5α chemically competent cell | Transgen | CD201 |
| *Saccharopolyspora erythraea* NRRL2338(WT) | DSM 40517 | AM420293.1 |
| Ab (Erythromycin high producing strain) | Ye’s Lab | Ye’s Lab |
| Ab∆*ery* | This study | N/A |
| Ab∆*ery*::AsCHS-RgTAL-Sc4CL | This study | N/A |
| WT::AsCHS-RgTAL-Sc4CL | This study | N/A |
| **Recombinant DNA and Plasmids** |  |  |
| ery-sgRNAII | This study | N/A |
| KOery-UHA | This study | N/A |
| KOery-DHA | This study | N/A |
| Pj23119-AsCHS | This study | N/A |
| PermE-RgTAL | This study | N/A |
| PkasO-Sc4CL | This study | N/A |
| pKECas9 (Liu et al., 2018) | Ye’s Lab | N/A |
| pSET152(kana) | Ye’s Lab | N/A |
| pUC57-*ery*-sgRNAII | This study | N/A |
| pKECas9-*ery*sgRNAII-UHA-DHA | This study | KO |
| pSET152-Pj23119-AsCHS-PermE-RgTAL-PkasO-Sc4CL | This study | N/A |

**Note**: N/A, not applicable. UHA, Upstream homologous arm; DHA, Downstream homologous arm

**Table S3.** Primers used in this study.

| **Primer name** | **Sequence (5'to3')** |
| --- | --- |
| **1H-*Xba*I-sgRNA-F** | gcgcgcggccgcggatcctctagaTTGACAGCTAGCTCAGTCCT |
| **2*ery*sgRNAII-R-O** | GTGGCGGGCGGTTTCGAGTTCACCCCCaagcttCAAAAAAAGCACCGACTCGGTGCC |
| **3*ery*Up-F-O** | GGCACCGAGTCGGTGCTTTTTTTGaagcttGGGGGTGAACTCGAAACCGCCCGCCAC |
| **4*ery*Up-R-O** | GAGACGGCGACGATGGTCACGCAGCTGAATTCcgggcaagtactggtccaacacgatc |
| **5*ery*Down-F-O** | gatcgtgttggaccagtacttgcccgGAATTCAGCTGCGTGACCATCGTCGCCGTCTC |
| **6H-*Hin*dIII-eryDown-R** | aaaacgacggccagtgccaagcttCCTTCAGTTCCGTCTGGTAGTGCTGCGCCAGCG |
| **pKECas9-test-F** | CTCGTGCTTCTTGTCCTCCTCCACC |
| **pKECas9-test-R** | cgccagggttttcccagtcacgac |
| ***ery*HKO-tF** | CATCTCCACCGCGTCCCACTTCCCGAAC |
| ***ery*HKO-tR** | GGTCGGGTAGTGGATGAGGGTCTGGA |
| **1H-*Xba*I-Pj23119-AsCHS-F** | gccaagcttgggctgcaggtcgactctagaTTGACAGCTAGCTCAGTCCTAGGTATAAT |
| **2AsCHS-R-O** | cggacactcgcatgcatactagAAAAGGCCATCCGTCAGGATGGCCTTCTTTAG |
| **3PermE-RgTAL-F-O** | ctaaagaaggccatcctgacggatggccttttCTAGTATGCATGCGAGTGTCCG |
| **4RgTAL-R-O** | GTCAAAGCAGAGACCGTTCGAATGTGAACActaggcaagcatcttaagaagcacgttg |
| **5PkasO-Sc4CL-F-O** | caacgtgcttcttaagatgcttgcctagTGTTCACATTCGAACGGTCTCTGCTTTGAC |
| **6H-*Eco*RV-Sc4CL-R** | cagctatgacatgattacgaattcgatatcATAAAAAACGCCCGGCGGCAACCGAGCG |
| **M13-F** | CGCCAGGGTTTTCCCAGTCACGAC |
| **M13-R** | AGCGGATAACAATTTCACACAGGA |
| **Q-1801-F** | GCTCCGCTGCAAACTCTGG |
| **Q-1801-R** | AGGTCTTGGCGCCCTTCTT |
| **Q-0713-F** | CCGATGGACCACGAGCAGTT |
| **Q-0713-R** | GCCAGGACAAGGCGGGAGAT |
| **Q-0714-F** | CCCTGATGTCGTACCTGGTCA |
| **Q-0714-R** | CCGAGGCAGTCGGCGTAGTCG |
| **Q-0715-F** | ACCGCAAGCCTGCCTTCCCCTA |
| **Q-0715-R** | CGAGCCCGCCCGACATGTACAT |
| **Q-0716-F** | GTGGGCGCTGAACAACAACTGG |
| **Q-0716-R** | GCGAACGTCATCGAAGGCATGA |
| **Q-0717-F** | CCCTGCTGGTCAAGGACATCG |
| **Q-0717-R** | CACATAGGCCGGTCGGAACT |
| **Q-0718-F** | ACGCCGTCACCTGCATGTTCAG |
| **Q-0718-R** | GCCACGACCCAGTGGATCTCCA |
| **Q-0719-F** | CAACATTCGTTTGGTGGATTT |
| **Q-0719-R** | GTTCTCGGTGTAGGTGGGGTC |
| **Q-0720-F** | CGCCGCCCGTGTTGCTCTA |
| **Q-0720-R** | CCTCGTCGGTGGCTTTGC |
| **Q-0721-F** | CGAGTGCGATGCGGTGTTG |
| **Q-0721-R** | CCTGCGAGTGCCCTATGACG |
| **Q-0722-F** | GTCGGGGTGGACCTGGGTGTGA |
| **Q-0722-R** | TCTTGGCTTTGCGGCGGTTGTT |
| **Q-0723-F** | GGTTGTCACACCCCTCATCA |
| **Q-0723-R** | CACGCCCCGGAATCCCGTTC |
| **Q-0724-F** | GGGTTCTCCCTCGATCTCGT |
| **Q-0724-R** | GGCTTGTCCCCCTGTGTCCT |
| **Q-0725-F** | GGCGACCCTTACCCGATGCTG |
| **Q-0725-R** | GCGAAGCTCTCCGCCAGTTCC |
| **Q-0726-F** | CCGAGTGGCTCACCTGGACGCT |
| **Q-0726-R** | ACCACGGACGGCCCGTTGTAGT |
| **Q-0727-F** | GGCAAGGTCTCCTACGTCGGCT |
| **Q-0727-R** | CCGCCAGGTTGTACAGGCACTG |
| **Q-0728-F** | GAAGCAGAAGTCAGCGTTGCA |
| **Q-0728-R** | TCCAGTAGCCGAGGTTGATGTAG |
| **Q-0729-F** | CACAGCATGGGCGCGTTGATC |
| **Q-0729-R** | GCTCGTCCACCAGACCGTCGT |
| **Q-0730-F** | CCTACCTGCTGCTCACCCACC |
| **Q-0730-R** | ACCGCCGATCTCCACCTCCTC |
| **Q-0731-F** | GCCTCGACCAAGGGCAACGT |
| **Q-0731-R** | GCCTCGACCAAGGGCAACGT |
| **Q-0732-F** | ATGCCGTGGCTGGACAAGACC |
| **Q-0732-R** | CCGAGAAGATGCCCTCCGAGTAG |
| **Q-0733-F** | ATCGGCAGCACCCCAACCAGA |
| **Q-0733-R** | CGGAAAGCTTCTCCCGCAACG |
| **Q-0734-F** | CAGCACTACCAGACGGAACTGAAGGAC |
| **Q-0734-R** | TCGCAGCGGAGGACGAAGAGG |
| **Q-AsCHS-F** | GAGCCGCCGCCATTATTGTA |
| **Q-AsCHS-R** | CGCCGTCGCTGTCTGGTAGA |
| **Q-RgTAL-F** | ACGCCGTAAACCGACATAGA |
| **Q-RgTAL-R** | GGTTACTCAGGTGGACATCGTG |
| **Q-Sc4CL-F** | GGCACTGCATAGCCCGAATA |
| **Q-Sc4CL-R** | GGACTAACGGTAACAATCCAACG |

**Note:** H indicates the homologous recombination sequence on the plasmid. The underlined sequences
are restriction endonuclease sites.

**TABLE S4.** The data of *ery* BGC transcriptional analysis in Ab and Ab∆*ery*.

| **SACE** | **C_T_ value** | | | | | | **Transcript level** | | | | | |
| --- | --- | --- | --- | --- | --- | --- | --- | --- | --- | --- | --- | --- |
|  | **Ab** | | | **Ab∆*ery*** | | | **Ab** | | | **Ab∆*ery*** | | |
| **0713** | 29.76 | 30.34 | 30.68 | 33.00 | 32.91 | 32.71 | 0.920 | 0.985 | 1.103 | 0.208 | 0.221 | 0.253 |
| **0714** | 30.04 | 30.07 | 30.35 | 32.94 | 32.94 | 32.39 | 1.053 | 1.011 | 0.939 | 0.201 | 0.200 | 0.293 |
| **0715** | 27.75 | 27.64 | 27.68 | 30.62 | 30.89 | 30.80 | 0.928 | 1.070 | 1.007 | 0.182 | 0.150 | 0.160 |
| **0716** | 25.48 | 25.28 | 25.40 | 28.53 | 28.42 | 28.48 | 0.937 | 1.079 | 0.989 | 0.047 | 0.051 | 0.048 |
| **0717** | 25.34 | 25.09 | 25.16 | 27.28 | 27.42 | 27.24 | 0.908 | 1.077 | 1.023 | 0.098 | 0.089 | 0.100 |
| **0718** | 25.60 | 25.28 | 25.51 | 27.49 | 27.76 | 27.65 | 0.907 | 1.137 | 0.969 | 0.102 | 0.084 | 0.091 |
| **0719** | 24.61 | 24.54 | 24.63 | 29.78 | 29.82 | 29.56 | 1.029 | 1.015 | 0.957 | 0.038 | 0.037 | 0.044 |
| **0720** | 26.95 | 26.80 | 26.97 | 31.46 | 31.47 | 31.59 | 0.857 | 1.051 | 1.110 | 0.059 | 0.059 | 0.054 |
| **0721** | 28.30 | 28.22 | 28.26 | 31.08 | 31.04 | 31.06 | 0.974 | 1.028 | 0.999 | 0.059 | 0.060 | 0.059 |
| **0722** | 25.74 | 25.72 | 25.98 | 29.46 | 29.49 | 29.46 | 1.052 | 1.068 | 0.890 | 0.033 | 0.032 | 0.033 |
| **0723** | 25.00 | 25.25 | 25.50 | 30.13 | 29.85 | 29.47 | 0.997 | 0.977 | 1.026 | 0.047 | 0.057 | 0.074 |
| **0724** | 29.89 | 29.98 | 29.81 | 34.72 | 34.11 | 34.47 | 0.821 | 1.137 | 1.072 | 0.043 | 0.066 | 0.052 |
| **0725** | 31.89 | 31.79 | 32.28 | 34.87 | 34.55 | 34.43 | 1.068 | 1.148 | 0.816 | 0.056 | 0.070 | 0.076 |
| **0726** | 29.04 | 29.08 | 29.27 | 32.76 | 33.18 | 32.83 | 0.922 | 0.814 | 1.333 | 0.099 | 0.074 | 0.095 |
| **0727** | 28.03 | 27.77 | 27.84 | 29.94 | 29.74 | 29.45 | 0.852 | 0.956 | 1.229 | 0.294 | 0.358 | 0.373 |
| **0728** | 26.56 | 26.61 | 26.60 | 29.74 | 29.97 | 29.35 | 0.949 | 1.030 | 1.023 | 0.138 | 0.118 | 0.181 |
| **0729** | 29.37 | 29.49 | 29.48 | 33.24 | 33.70 | 33.52 | 1.058 | 0.969 | 0.975 | 0.030 | 0.022 | 0.037 |
| **0730** | 26.75 | 27.29 | 26.71 | 28.04 | 28.14 | 28.03 | 1.122 | 0.774 | 1.152 | 0.059 | 0.055 | 0.059 |
| **0731** | 26.81 | 27.09 | 26.93 | 27.81 | 27.73 | 27.85 | 1.021 | 1.103 | 0.888 | 0.344 | 0.364 | 0.335 |
| **0732** | 27.13 | 27.05 | 27.35 | 30.29 | 30.06 | 29.85 | 1.033 | 1.092 | 0.886 | 0.015 | 0.017 | 0.020 |
| **0733** | 23.49 | 23.57 | 23.43 | 26.94 | 27.02 | 26.89 | 1.006 | 0.951 | 1.045 | 0.038 | 0.036 | 0.040 |
| **0734** | 27.85 | 28.00 | 28.59 | 30.59 | 30.81 | 30.90 | 0.930 | 1.142 | 0.942 | 0.209 | 0.244 | 0.228 |

**TABLE S5.** Phanata Max Super-Fidelity PCR reaction system.

| Components | Volume(µL) |
| --- | --- |
| Distilled Mini-Q H_2_O | 20.0 |
| 2×Phanata Max Buffer | 25.0 |
| dNTP Mix (10 mM each) | 1.0 |
| P_F_ | 1.0 |
| P_R_ | 1.0 |
| Phanata Max Super-Fidelity DNA Polymerase | 1.0 |
| Template | 1.0 |
| Total | 50.0 |

**Procedure:** 95°C 5min; 95°C 15s, 60°C 15s, 72°C 3min; 34 cycles; 72°C 5min; 12°C hold.

**TABLE S6.** Hieff One Step Cloning reaction system.

| Components | Volume(µL) |
| --- | --- |
| 2×Hieff Clone Enzyme premix | 10.0 |
| DNA fragment | 8.5 |
| Linear plasmid | 1.5 |
| Total | 20.0 |

**Procedure:** 50°C 30min, on ice 10 min.

**TABLE S7.** Hieff PCR Master Mix reaction system.

| Components | Volume(µL) |
| --- | --- |
| Distilled Mini-Q H_2_O | 7.5 |
| 2×Hieff PCR Master Mix (With Dye) | 10.0 |
| P_F_ | 0.75 |
| P_R_ | 0.75 |
| Template | 1.0 |
| Total | 20.0 |

**Procedure:** 95°C 5min; 95°C 15s, 60°C 15s, 72°C 3min; 31 cycles; 72°C 5min; 12°C hold.

**TABLE S8. Erythromycin Bioactivity Assay**

| Strains | Bioactivity (µg/mL) | | |
| --- | --- | --- | --- |
| Ab (Control) | 17.03 | 18.12 | 18.97 |
| AbΔery-1 | 7.64 | 8.78 | 7.94 |
| AbΔery-2 | 3.15 | 4.09 | 4.00 |
| AbΔery-3 | 8.85 | 11.32 | 9.34 |
| AbΔery-4 | 2.89 | 2.81 | 3.10 |
| AbΔery-5 | 4.03 | 4.06 | 4.22 |
| AbΔery-6 | 3.03 | 2.66 | 2.68 |
| AbΔery-7 | 6.76 | 7.35 | 7.24 |
| AbΔery-8 | 3.25 | 3.10 | 3.51 |
| AbΔery-9 | 5.12 | 5.70 | 5.08 |
| AbΔery-10 | 3.85 | 3.91 | 3.70 |
| AbΔery-11 | 5.04 | 5.36 | 4.93 |
| AbΔery-12 | 4.19 | 4.49 | 4.81 |

Note: Screening Ab∆*ery* BGC recombinant strains by bioactivity assay. Ab∆ery and the control Ab were cultured in TSB shake flasks for 72 h, and the erythromycin content in the fermentation broth was analyzed by the biological activity method.

**SUPPLEMENTARY REFERENCES**

Bibb, M.J., Janssen, G.R., and Ward, J.M. (1985). Cloning and analysis of the promoter region of the erythromycin resistance gene (ermE) of Streptomyces erythraeus. *Gene* 38, 215-226. doi: 10.1016/0378-1119(85)90220-3

Gao, B., Wang, X., Liu, X., Shi, S., and Tu, P. (2015). Rapid preparation of (methyl)malonyl coenzyme a and enzymatic formation of unusual polyketides by type III polyketide synthase from Aquilaria sinensis. *Bioorg. Med. Chem. Lett.* 25, 1279-1283. doi: 10.1016/j.bmcl.2015.01.045

He, H., Guosong, Z., Weihong, J., Haifeng, H., and Yinhua, L. (2015). One-step high-efficiency CRISPR/Cas9-mediated genome editing in Streptomyces. *Acta Bioch. Bioph. Sin.* 47, 231-243. doi: 10.1093/abbs/gmv007

Hsiao, N., and Kirby, R. (2008). Comparative genomics of Streptomyces avermitilis, Streptomyces cattleya, Streptomyces maritimus and Kitasatospora aureofaciens using a Streptomyces coelicolor microarray system. *Antonie van Leeuwenhoek* 93, 1-25. doi: 10.1007/s10482-007-9175-1

Liu, Y., Ren, C., Wei, W., You, D., and Yin, B., et al. (2019). A CRISPR-Cas9 strategy for activating theSaccharopolyspora erythraea erythromycin biosynthetic gene cluster with knock-in bidirectional promoters. *ACS Synth. Biol.* 8, 1134-1143. doi: 10.1021/acssynbio.9b00024

Liu, Y., Wei, W.P., and Ye, B.C. (2018). High GC content Cas9-Mediated Genome-Editing and biosynthetic gene cluster activation in saccharopolyspora erythraea. *ACS Synth. Biol.* 7, 1338-1348. doi: 10.1021/acssynbio.7b00448

Wang, W., Li, X., Wang, J., Xiang, S., and Feng, X., et al. (2013). An engineered strong promoter for streptomycetes. *Appl Environ Microbiol* 79, 4484-4492. doi: 10.1128/AEM.00985-13

Wu, J., Zhou, T., Du G, Zhou, J., and Chen, J. (2014). Modular optimization of heterologous pathways for de novo synthesis of (2S)-naringenin in Escherichia coli. *PLoS One* 9, e101492. doi: 10.1371/journal.pone.0101492
